# Anti-Kasha Behavior of 3-Hydroxyflavone and Its Derivatives

**DOI:** 10.3390/ijms222011103

**Published:** 2021-10-14

**Authors:** Ka Wa Fan, Hoi Ling Luk, David Lee Phillips

**Affiliations:** 1 Department of Chemistry, University of Hong Kong, Hong Kong 999077, China; u3532679@connect.hku.hk; 2 Guangdong-Hong Kong-Macao Joint Laboratory of Optoelectronic and Magnetic Functional Materials, Hong Kong 999077, China

**Keywords:** anti-Kasha, ESIPT, 3-hydroxyflavone

## Abstract

Excited state intramolecular proton transfer (ESIPT) in 3-hydroxyflavone (3HF) has been known for its dependence on excitation wavelength. Such a behavior violates Kasha’s rule, which states that the emission and photochemistry of a compound would only take place from its lowest excited state. The photochemistry of 3HF was studied using femtosecond transient absorption spectroscopy at a shorter wavelength excitation (266 nm), and these new experimental findings were interpreted with the aid of computational studies. These new results were compared with those from previous studies that were obtained with a longer wavelength excitation and show that there exists a pathway of proton transfer that bypasses the normal first excited state from the higher excited state to the tautomer from first excited state. The experimental data correlate with the electron density difference calculations such that the proton transfer process is faster on the longer excitation wavelength than compared to the shorter excitation wavelength.

## 1. Introduction

Kasha’s rule states that, in any condensed phase, photon emission (fluorescence or phosphorescence) occurs in appreciable yields only from the lowest state of a given (singlet or triplet) multiplicity, irrespective of the initial photoexcited state [1,2]. However, a number of exceptions to this rule have been observed, including some compounds that exhibit proton transfer on the excited state [3]. Excited-state intramolecular proton transfer (ESIPT) is one of the fundamental reactions in chemistry and biology [4,5]. It is a process in which the photo-excited molecule can relax through the transfer of a proton within the molecule. Even though the ESIPT process may have an extremely fast nature, two fluorescence emission bands from the reactant normal (N) and product tautomer (T) states can be observed in some cases. This results in interest from researchers who are seeking fluorescent probes, and, in this regard, the 3-hydroxyflavone (3HF) molecular family is one that has attracted considerable interest. This is because such dual emissions of 3HF are sensitive to the properties of the surrounding environment such as polarity and H-bond donor and acceptor ability of the condensed phase environment [6,7], and such unique properties have already found a variety of applications for studying polymers [8], host-guest complexes [9,10], reverse micelles [11,12], model lipid membranes [13], biomembranes [14] and proteins [15].

**Scheme 1:**
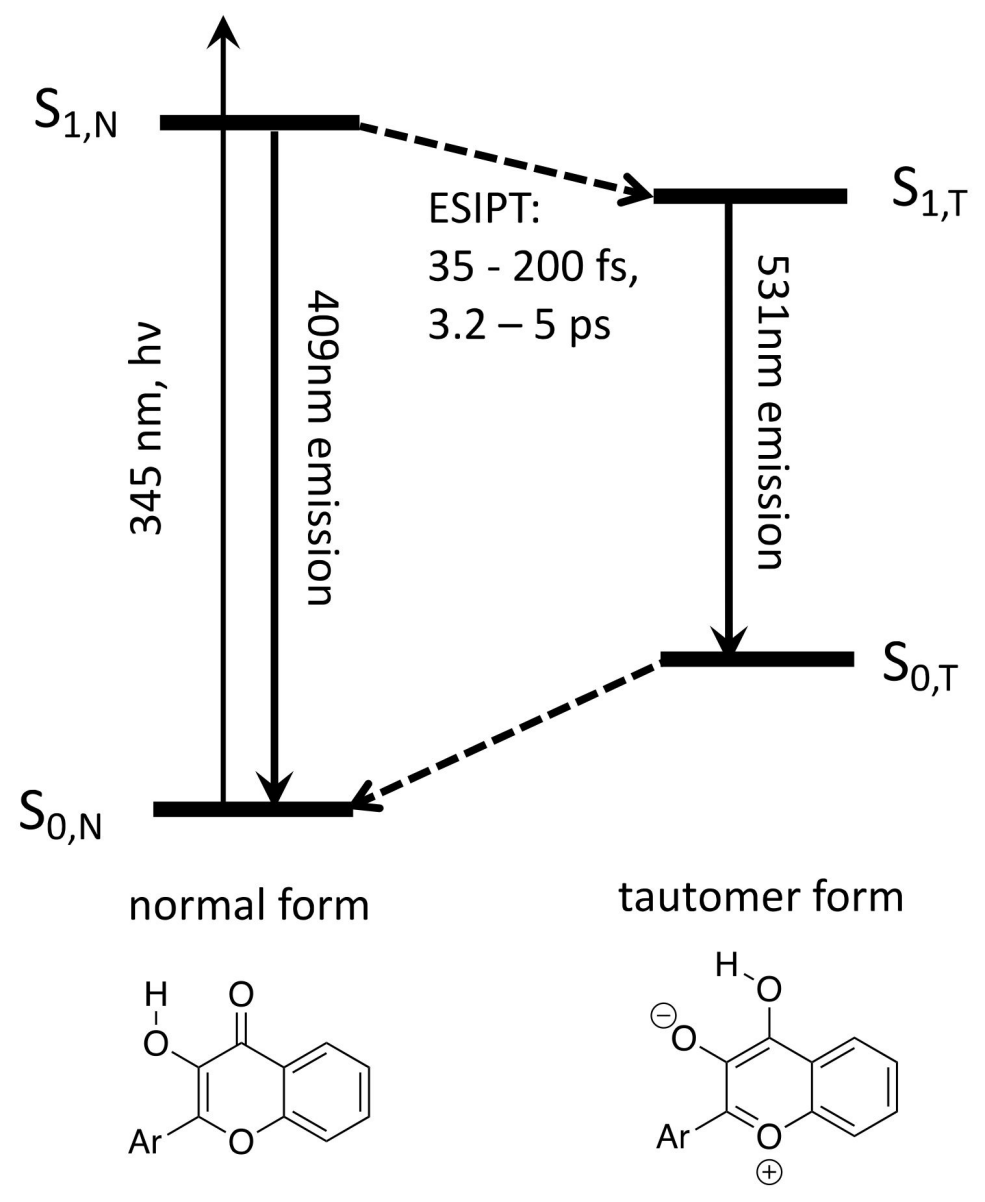
Photocycle of 3HF with lower energy UV excitation. After initially populating the excited state S_1,N_, ESIPT occurs with time constant 35–200 fs and 3.2–5 ps to form the excited tautomer state [16] and decay back to the ground state S_0,T_ followed by an ultrafast ground state back to normal form S_0,N_. Dual fluorescence emission bands were observed from S_1,N_ and S_1,T._ [17,18].

3HF begins with an excitation to an excited state of the reactant normal form (S_1,N_) which rapidly undergoes ESIPT, forming the excited state of the tautomer form (S_1,T_). (Scheme 1). Two transient absorption bands at around 450 and 610 nm were observed in previous studies and can be assigned to S_1,T_, considering their equal lifetimes and computation results [16]. The decay of S_1,T_ to the tautomeric ground state S_0,T_ either through non-radiative decay or fluorescence is directly followed by an ultrafast ground state back proton transfer completing the photocycle.

The positions of the transient absorption bands and the time constant of ESIPT depend on the solvent medium. Studies in various solvents showed two different sets of time scales for proton transfer. In non-polar environments such as methylcyclohexane, ESIPT occurs on an ultrafast timescale (<200 fs) [19,20,21] while in polar environments such as acetonitrile, it occurs with two timescales, an ultrafast (<200 fs) and a picosecond one [16,22,23]. These findings result in the suggestion of there being two different species of 3HF molecules in the excited state, one with an intact intramolecular hydrogen bond and the other with a weakened intramolecular hydrogen bond due to the interactions with surrounding solvent molecules, both undergoing ESIPT but with different time constants. The slower picosecond time constant was suggested to arise from intermolecularly bonded 3HF-solvent aggregates that must be perturbed first for the proton transfer in order to proceed, whereas the ultrafast time constant arises from the unperturbed 3HF molecules with an intact intramolecular hydrogen bond that is ready for immediate proton transfer.

**Scheme 2:**
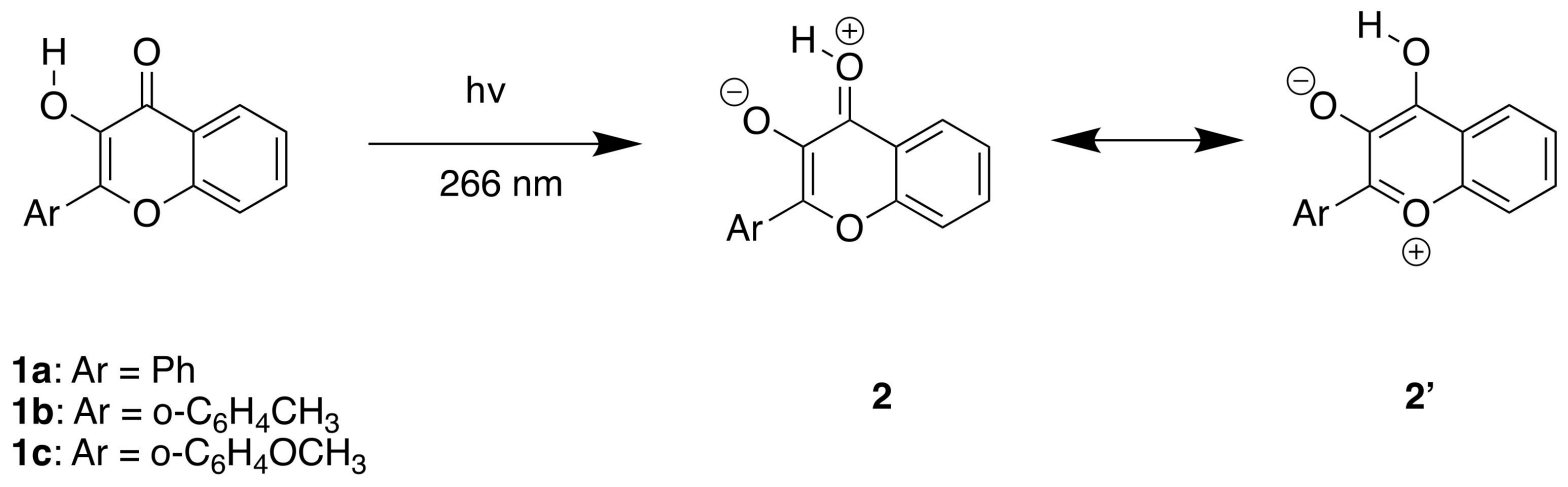
Excited state intramolecular proton transfer reaction for 3HF and its derivatives investigated in this study.

Dual fluorescence bands were observed in the excitation of 3HF and can be attributed to the two excited states: (1) S_1,N_ produces a blue fluorescence band at 409 nm and (2) S_1,T_ produces a green fluorescence band at 531 nm (Scheme 1). However, it was observed that there is a dependence of the intensity ratio of these blue and green fluorescence bands of 3HF on the excitation wavelength [17,18]. This ratio decreases with smaller excitation wavelengths. This would show that the higher excitation states definitely play an important role in photochemistry. Moreover, Tomin compared the photochemistry of 3HF with two different excitation wavelength of 300 nm and 340 nm, respectively [24]. Their results show a faster ESIPT rate reaction with a shorter wavelength excitation, and there were debates about the possibility of ESIPT of higher excited states [25,26]. Unfortunately, most of previous ultrafast experimental work focused on the establishment of the photocycle found with lower energy excitation relative to the first excited state [16,27]. Therefore, the ultrafast UV-Vis transient absorptions were performed on these 3HF derivatives along with quantum chemical calculations to understand the photochemical mechanism at a higher excited state in order to help explain why there is such a dependence of the intensity ratio of the fluorescence bands. Moreover, both the spectroscopic and the solvatochromic properties of the 3HF family of molecules can be finely tuned by different chemical substituents so that these fluorophores can be optimized for a particular application. For instance, we are interested in studying the effect of such substituents on photochemistry (Scheme 2).

## 2. Results

### 2.1. Experimental Results

**Figure 1:**
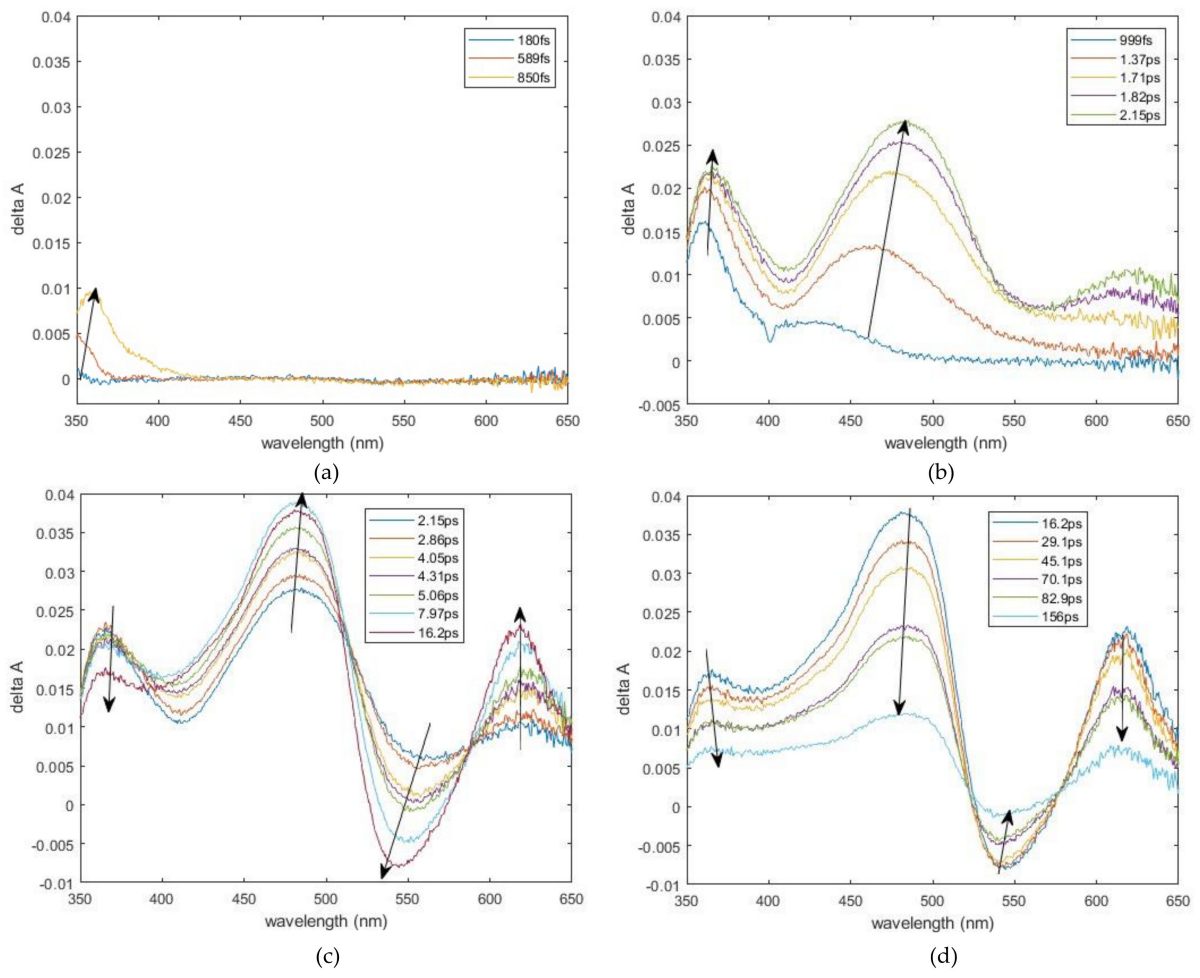
Transient UV-vis absorption spectra produced by fs laser excitation of 1b in acetonitrile (λ_max_ = 266 nm) with different time frames: (**a**) <1 ps; (**b**) 1–2 ps; (**c**) 2–16 ps; and (**d**) 16–156 ps.

Figure 1 shows the transient UV–Vis absorption spectra of 1b in acetonitrile with 266 nm excitation. The femtosecond transient absorption spectra show an initial appearance of a 362 nm band, and it reaches its maximum intensity at ~2 ps. (Figure 1b). At around 1 ps, a distinct positive band develops at around 482 nm, and it is followed by another positive band at 617 nm and one negative band at around 540 nm at 2 ps. (Figure 1c). The 482 nm and 617 nm bands were assigned previously to the tautomer excited state absorption, while the 540 nm negative band was assigned as the tautomer simulated emission [16]. In the meantime, the 361 nm band starts to decay, and the other three bands join in the decay after 16 ps (Figure 1d).

Table 1 shows the fitted exponential rise and decay lifetime constants for the different transient absorption bands. All the rising and decay lifetimes of 482 nm, 540 nm and 617 nm were fitted with a mono-exponential with a similar rising lifetime of 2–4 ps and a decay lifetime of ~110 ps. The rise of 361 nm band is beyond the time resolution of our system and should be <100 fs, while the decay lifetime of 361 nm band was fitted with biexponential with a lifetime of 6 ps and 100 ps. The observed rising lifetimes for the three longer wavelength bands are similar to what was observed with the lower excitation wavelength (~5 ps) [16,27], but the femtosecond components observed for these bands in the lower excitation wavelength were not observed with higher excitation. All of these results point towards a different photochemical mechanism with higher excitation wavelength than the lower wavelength.

### 2.2. Computation Results

In order to better understand and interpret the experimental results obtained in this study, we performed density functional theory calculations on some singlet excited states of 1b and its tautomer. Table 2 shows the results obtained from time-dependent density functional theory (TDDFT) calculations of 1b in acetonitrile at the level of theory of B3LYP/6-311G(d) with a universal solvation model (SMD). These results show that with a 266 nm excitation, 1b would mainly populate the fourth excited state (S_4_) due to its relatively larger oscillator strength rather than the S_1_ state.

**Figure 2:**
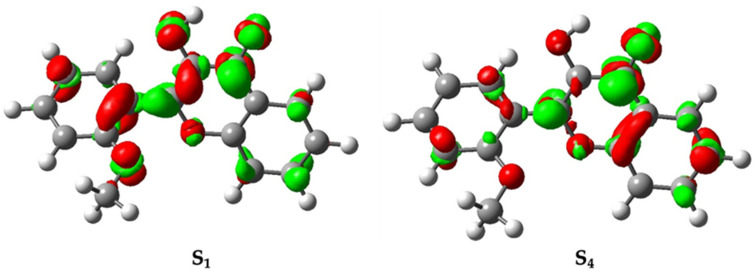
The electron density difference plots for the S_1_ and S_4_ states for 1b as calculated relative to the S_0_ ground state at the TD-B3LYP/6-311G(d) level of theory with SMD models in acetonitrile. The green contours depict the accumulation of electron density in the excited state, and the red contours illustrate the depletion of electron density from the S_0_ ground state. The isocontour value is ±0.0024 au.

The difference between S_1_ and S_4_ states can be observed with the electron difference plot of these states relative to the ground state (Figure 2). These two states differ by the position of electron density depletions, with the S_1_ state observed from the phenyl ring while the S_4_ state is observed from the heterocyclic ring. Nevertheless, both states would provide the accumulation of electron density at the π* orbital of the C=O bond, which would promote proton transfer.

**Figure 3:**
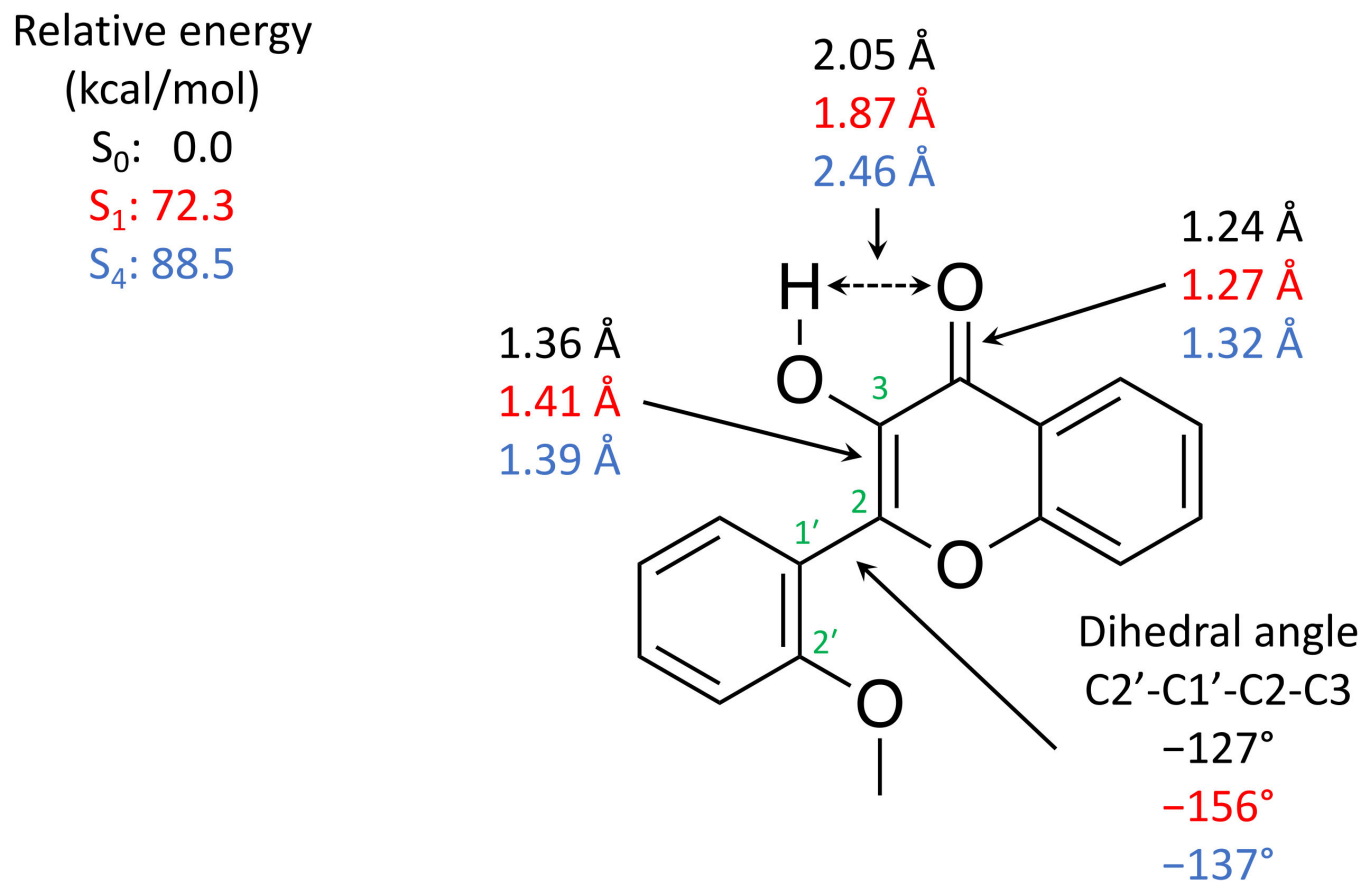
Relative energies (in kcal/mol) and representative bond lengths in angstroms and dihedral angle in degrees for the optimized ground state (S_0_ in black), first excited state (S_1_ state in red) and fourth excited state (S_4_ state in blue), respectively, from Franck–Condon geometry of 1b at the TD-B3LYP/6-311G(d) level of theory with SMD models in acetonitrile.

Excited state optimizations were also performed for the S_1_ and S_4_ states from the Franck–Condon geometry of 1b. The corresponding relative energies and representative geometric parameters are shown in Figure 3. The significance of these structures will be discussed in the next section.

## 3. Discussion

### 3.1. Band Assignments

Since the femtosecond UV absorption spectra for the 3HF derivatives performed provided similar results (see Figure S4, Tables S1 and S2), the following discussion will be focused on the results for 1b. From the femtosecond transient UV absorption spectra of 1b with higher energy excitation (e.g., 266 nm), we observed three distinct absorption bands (361 nm, 482 nm and 617 nm) and one emission band (540 nm). The two absorption bands, 482 nm and 617 nm, can be assigned as the absorption bands of the first excited state of the tautomer (S_1,T_), while the 540 nm negative band can be assigned as the stimulated emission band of S_1,T_. These observed bands are at similar positions as observed in the previous studies performed with lower energy excitation (e.g., 345 nm) [16,27]. However, there is an absence of femtosecond components in these bands in our study. These femtosecond components were previously assigned as the fast ESIPT process of the unperturbed molecule with intact intramolecular hydrogen bonding. The reason for the absence of these femtosecond components is likely due to the population of the higher excited state (S_n_) instead of the S_1_ state. The molecules need to rearrange and relax to a lower excited state for the ESIPT process, which would take a few picoseconds to occur as observed in the new data. This is similar to what was observed in the S_1_ state, where the intermolecularly bonded reactant solvent aggregates, which must be perturbed first for the proton transfer to proceed.

**Scheme 3:**
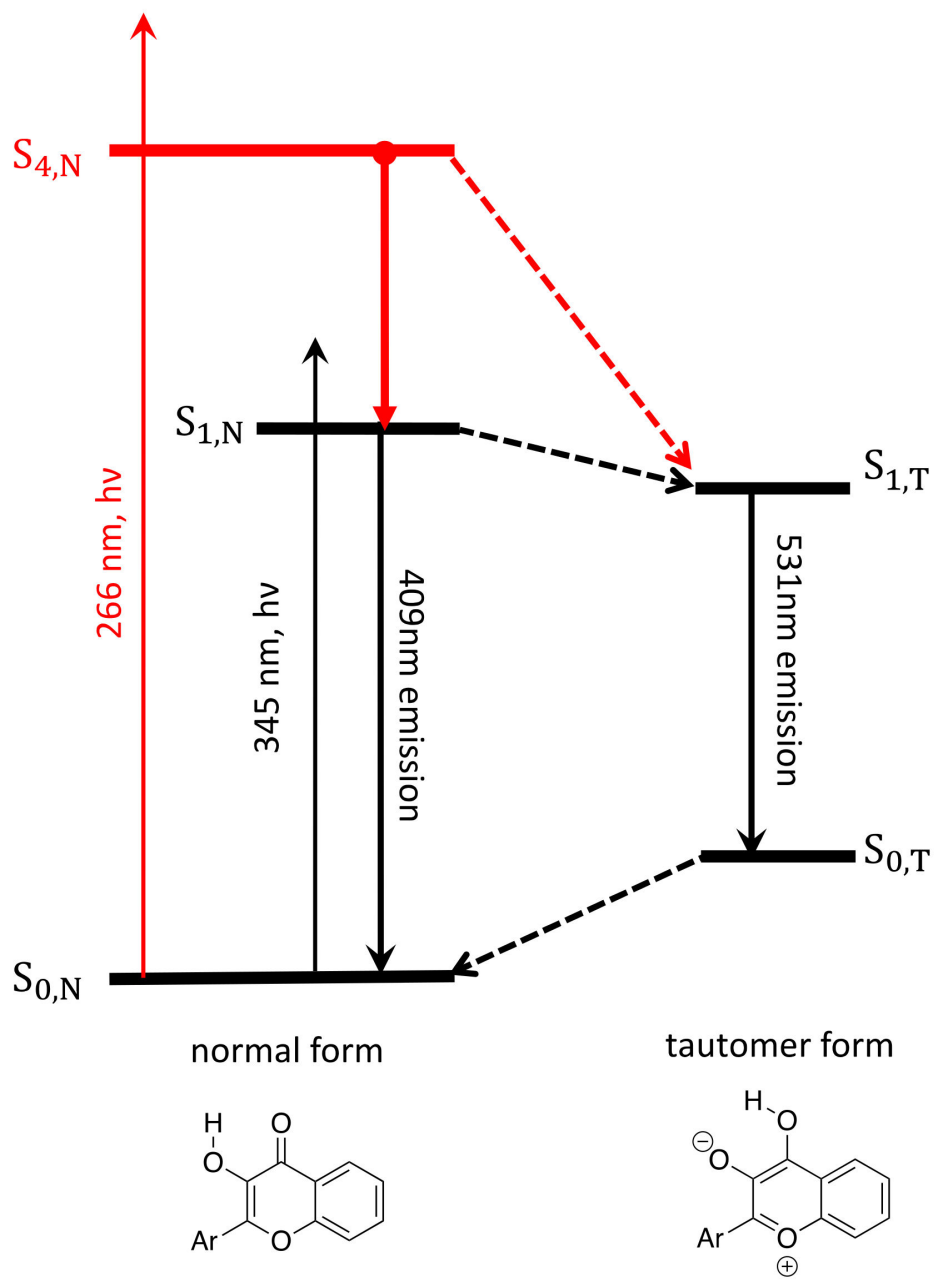
Photochemistry of 3HF with higher energy excitation in red compared to the lower excitation energy in black.

The newly observed absorption band at 361 nm has only been observed with higher energy excitation. There are four potential candidates, namely, the first excited state of the normal form S_1,N_, the first excited state of tautomer form S_1,T_, the fourth excited state of normal form S_4,N_ and the fourth excited state of tautomer form S_4,T_. One can opt out of the possibility of S_1,N_ because this is not observed in the previous studies [16,27] with lower energy excitation, and the lack of a femtosecond component in its decay than compared to ESIPT on the S_1_ state always includes an ultrafast component (<200 fs) [16,22,23] and such an ultrafast component should be within our detection limit of the system. This 361 nm band cannot be assigned as S_1,T_ since the 361 nm band possesses biexponential decay (6 ps and 100 ps), which is different from the mono-exponential decay observed in the other absorption bands attributed to S_1,T_ (~110 ps). It is also unlikely to be the fourth excited state of the tautomer form S_4,T_ (Scheme 3) because one would expect the decay of S_4,T_ to be mono-exponential to S_1,T_ instead of biexponential. Hence, the 361 nm band is assigned to be the absorption band of the fourth excited state of the normal form (S_4,N_ in Scheme 3).

The decay of the 361 nm band consists of a faster component of 6 ps and a slower component of 100 ps. The faster decay component of 6 ps can be attributed to the ESIPT proceeding through the first excited normal state (S_1,N_) in a few picoseconds similar to what was previously observed in the lower energy excitation (~5ps) [16]. However, since there is a decrease in blue fluorescence intensity from the S_1,N_ state with the higher excitation wavelength [17,18], it is unlikely that all the molecules from the S_4,N_ state decay back to the S_1,N_ state. There must exist another pathway that has bypassed the formation of the S_1,N_ state and resulted in a decrease in blue fluorescence intensity. Hence, the slower component of 100 ps is assigned to be the ESIPT proceeding directly from S_4,N_ to S_1,T_, which can be further supported from the computational results in the next section.

### 3.2. Interpretation of Our Proposed Computational Model

Based on the TDDFT calculations, 266 nm excitation would excite the normal form to the fourth excited state (S_4_) instead of the first excited state observed in previous studies [16,27]. The Franck–Condon geometry is relaxed on the fourth excited state through the elongation of the C=O bond (Figure 3). This is consistent with the observation from the difference electron density plot with the accumulation of electron density at the π* of the C=O bond (Figure 2). The elongation of the C=O bond and accumulation of the electron density on the carbonyl oxygen are favorable observations for proton transfer reactions since the carbonyl oxygen becomes a better proton acceptor. However, there is an increase in the distance between the hydroxy hydrogen and the carbonyl oxygen from 2.05 Å in the ground state to 2.46 Å in the fourth excited state. This is different from the optimized structure on the first excited state where, instead, there is a decrease in such a distance from the ground state geometry to 1.87 Å. This geometric difference suggests that a longer period of time might be required for the molecule to rearrange for the proton transfer on the S_4,N_ state than in the S_1,N_ state. Moreover, by comparing difference electron density plots (Figure 2), there is a depletion of electron density on the hydroxy oxygen for the S_1,N_ state, which is absent in the S_4,N_ state. These observations suggest that the S_1,N_ state is more favorable for proton transfers than the S_4.N_ state because there should be a loss in the electron density for hydroxy oxygen and a gain in electron density for carbonyl oxygen in order to promote proton transfers from hydroxy oxygen to carbonyl oxygen. This would explain why the ultrafast proton transfer was not observed in the higher energy excitation and one would expect that the proton transfer from the S_4_ state would take a longer relaxation time period either through the relaxation back to S_1,N_ or a slower period directly to S_1,T._

The depletion of electron density on the hydroxy oxygen on S_1,N_ is a result of the depletion of electron density from the phenyl ring instead of the heterocyclic ring on the S_4,N_ state. From the optimized structures of the respective excited states (Figure 3), the phenyl ring and the heterocyclic ring would be more coplanar in the S_1,N_ state than in the ground state or the S_4,N_ state. Due to the conjugation along C2′-C1′-C2-C3, the C2-C3 bond would elongate in the S_1,N_ state (1.41 Å) relative to the ground state (1.36 Å) or the S_4,N_ state (1.39 Å). This more closely resembles the resonance structure 2′ (Scheme 2), which promotes proton transfer. This also suggests that the electron donating group that is substituted on the ortho/para position on the phenyl ring would assist such a conjugation and promotes proton transfer, which is consistent with what was observed in previous studies [16,28].

In order to account for the slightly faster growth lifetime of the tautomer peaks (S_1,N_) (~2–4 ps) observed compared to the decay of the S_4,N_ state (6 ps), it is likely that after the excitation to the fourth excited state at the Franck–Condon region, some of the molecules underwent internal conversion back to the S_1,N_ state instead of vibrationally relaxing in the S_4,N_ state. Hence, the 482 and 617 nm absorption peaks of S_1,T_ can be observed before the decay of the 361 nm peak, which is attributed to the vibrationally relaxed fourth excited state (S_4,N_).

For those molecules that were relaxed in the fourth excited state (S_4,N_), they would either decay back to the first excited state before the proton transfer or undergo proton transfer directly to S_1,T_. The proton transfer from the fourth normal excited state (S_4,N_) to the fourth excited state tautomer form (S_4,T_) is to occur unlikely because the computed energy of the relaxed S_4,N_ is significantly more stabilized from the Franck–Condon geometry on the fourth excited state (~14 kcal/mol) than compared to what it was in the relaxed first excited state (~10 kcal/mol). This would mean that the molecules would require overcoming a higher barrier for proton transfers within the same state. This is further supported by the proton transfer energy profile where the proton transfer barrier is higher in the S_4_ state than compared to that in the S_1_ state (Figure S1). These results suggest that the proton transfer within the fourth excited state is unlikely, and it is more likely to relax back to S_1,T_ instead.

Moreover, Tomin compared the photochemistry of 3HF with two different excitation wavelength of 300 nm and 340 nm, respectively [24]. Their results show a faster ESIPT rate reaction with shorter wavelength excitation. This supports the argument that additional proton transfer channel exists with a higher excitation wavelength, which is consistent with our observations with an even higher excitation wavelength (266 nm) and based on our difference electron density plots which suggest that such a proton transfer from higher excited state back to the S_1,T_ state is feasible.

### 3.3. The Long-Lived S_4,N_

The relatively long-lived S_4,N_ state is assigned to proton transfer relative to S_1,T_ (100 ps), which has similar decay lifetime of S_1,T_ (~113 ps). Such an assignment is needed because there exist two channels to populate S_1,T_. One comes from S_4,N_ and the second one comes from S_1,N_, which comes from the non-radiative decay of S_4,N_. Therefore, the absorption band of S_1,T_ can rise within picoseconds. The absence of new fluorescence bands indicates that S_4,N_ would not directly proceed to the ground state. Hence, it would undergo both internal conversion relative to S_1,N_ and proton transfer relative to S_1,T_. If one only considers the scenario with S_4,N_ decaying back to S_1,N_ internal conversion, it would fail to explain the observation of the decrease in fluorescence intensities relative to normal form and tautomer forms [17]. Thus, an additional channel for bypassing the S_1,N_ state from the higher excited state must exist. Therefore, the mechanism in Scheme 3 is suggested for the photochemistry of 3HF with a relatively long decay for the proton transfer from the S_4,N_ state to S_1,T_ state.

In conclusion, we have demonstrated, by using ultrafast time-resolved spectroscopy, that the photochemistry of 3-hydroxyflavone is wavelength dependent. With a higher energy excitation, the ESIPT is not ultrafast, as is observed in the lower energy excitations. The ESIPT process takes a few picoseconds because it takes time for perturbation of the molecules to lower excited states before the proton transfer can proceed. Our computational results also suggest the major differences between the two excited states such that the lower excited state is more favorable for proton transfers than the higher excited state, which is a result from the phenyl ring π to π* transition in the lower excited state instead of the heterocyclic ring π to π* transition in the higher excited state. Nevertheless, there still exists a proton transfer pathway that bypasses the normal form in lower excited states to account for the reduced intensity of fluorescence from the normal state with higher energy excitations.

## 4. Materials and Methods

### 4.1. Synthesis

The chemicals were purchased from TCI (Shanghai) Development Co., Ltd., Shanghai, China and used without further purification. All the compounds were synthesized following the reported literature methods [29]. The products were characterized by NMR and reported in the Supplementary Materials.

### 4.2. Femtosecond Transient Absorption Experiment

The Ti:Sapphire regenerative amplifier laser system was used in the femtosecond transient absorption measurement. Details of the setups can be found [30]. The samples were dissolved in spectroscopic grade acetonitrile in 2 mm UV cell prepared by an approximate absorbance of 1.0 at 266 nm. The pump source of 266 nm came from a third harmonic of 800 nm, and the 350 nm–650 nm probe source was generated by an 800 nm pass through CaF_2_. The experiment had a 120 fs duration between the excitation pulse and probe pulse.

### 4.3. Computational Methods

All of the calculations were performed using the Gaussian 16 suite [31]. The ground state geometry of 1b was optimized at the B3LYP level with a 6-311G(d) basis set with a universal solvation model (SMD) in acetonitrile. Vertical excitations were computed by using the time dependent TD-B3LYP level of theory at the optimized structure with 50 states. In order to characterize the excited states, we computed different electronic density plots (between S_0_ and S_1_–S_5_ states), as described in previous reports [32,33]. The first and fourth singlet excited states of 1b were optimized from Franck–Condon geometry. All geometries were confirmed to be at the minimum by calculating the second derivatives.

## Figures and Tables

**Table 1:** Transient kinetics observed at various probe wavelengths of 1b.

Transient Absorptions (nm)	Growth Lifetime	Decay Lifetime
	τ_r_, ps (A_r_)	τ_1_, ps (A_1_) τ_2_, ps (A_2_)
361	<0.1	6.5 ± 0.6 (4.3 × 10^−3^) 100 ± 2.5 (1.4 × 10^−2^)
482	2.6 ± 0.4 (4.8 × 10^−2^)	113 ± 1.3 (3.8 × 10^−2^)
540	4.4 ± 0.1 (−1.8 × 10^−2^)	113 ± 2.4 (−1.0 × 10^−2^)
617	3.1 ± 0.2 (2.5 × 10^−2^)	111 ± 1.9 (2.2 × 10^−2^)

**Table 2:** Vertical excitation energies, oscillator strengths, and the dominant occupied and virtual orbitals contributing to the five lowest energy singlet excitations of 1b at the level of theory of TD-B3LYP/6-311G(d), with universal solvation model (SMD) model in acetonitrile.

Excited State	Energy (eV) (Wavelength (nm))	Major Character (% Contributions)	Oscillator Strength
S_1_	3.60	HOMO → LUMO (90%)	0.2858
	(345)		
S_2_	3.92	HOMO-1 → LUMO (89%)	0.0453
	(317)		
S_3_	4.18	HOMO-3 → LUMO (44%)	0.0017
	(296)	HOMO-4 → LUMO (39%)	
S_4_	4.44	HOMO-2 → LUMO (76%)	0.1108
	(279)		
S_5_	4.63	HOMO → LUMO + 1 (74%)	0.0323
	(268)		

## Data Availability

Not applicable.

## Supplementary Materials

The following are available online at https://www.mdpi.com/article/10.3390/ijms222011103/s1.
