# Supplementary material for: Anti-Kasha Behavior of 3-Hydroxyflavone and Its Derivatives
Source: Int J Mol Sci. 2021 Oct 14;22(20):11103. doi: 10.3390/ijms222011103 (PMC8540728; doi:10.3390/ijms222011103)
Supplement: Supplementary file 1 [file ijms-22-11103-s001.zip › ijms-1357282-supplementary.pdf]

Supplementary Materials

Anti-Kasha Behavior of 3-Hydroxyflavone and Its Derivatives

1. General information

All chemical purchase from TCI Shanghai and use without further purification. The synthesis follows the procedure detiled in Org. Lett. 2012, 14, 6, 1576–1579. To a 500ml round-bottom flask equipped with a magnetic stir bar were added 2′-Hydroxyacetophenone (2.0ml, 16.6mmol), 2-Methoxybenzaldehyde (2.0ml, 16.6mmol), NaOH (2.0g,50mmol) and methanol (100ml). The color of the mixture changed from a pale yellow to a purple color during reflux conditions (about 3 hours). The mixture was cooled down to room temperature and added 100 ml of 0.5N NaOH solution with 10ml 50%H_2O_2. The product was extracted with ethyl acetate and dried by $MgSO_4$. Then, the solvent was removed by using an evaporator rotary.

3-hydroxy-2-(2-methoxyphenyl)-4H-chromen-4-one

1H NMR(400 MHz,DMSO):δ/ ppm =8.94(singlet,1H), 8.15(dd,1H),7.78(td,1H),7.63(d,1H), 7.4(m,3H),7.20(d,1H),7.10(td,1H) , 3.80(singlet,3H)

13C NMR(101 MHz,DMSO): δ/ ppm= 173.2, 157.6, 155.4, 147.6, 139.6, 134.0, 132.2, 131.5, 125.3, 125.0, 122.4, 120.6, 120.3, 118.9,112.4,56.2

3-hydroxy-2-o-tolyl-4H-chromen-4-one

1H NMR(400 MHz,DMSO):δ/ ppm =9.15(singlet,1H), 8.17(dd,1H),7.80(td,1H),7.66(d,1H), 7.56(dd,1H), 7.47(td,1H),7.43(dd,1H),7.36(t,1H),7.32(td,1H), 2.31(singlet,3H)

13C NMR(101 MHz,DMSO): δ/ ppm= 173.3, 155.45, 149.0, 139.2 , 137.5 , 134.1, 131.1, 130.8, 130.4, 130.3, 126.0, 125.4, 125.1, 122.4, 118.9, 20.0

2. NMR Spectra

3-hydroxy-2-(2-methoxyphenyl)-4H-chromen-4-one

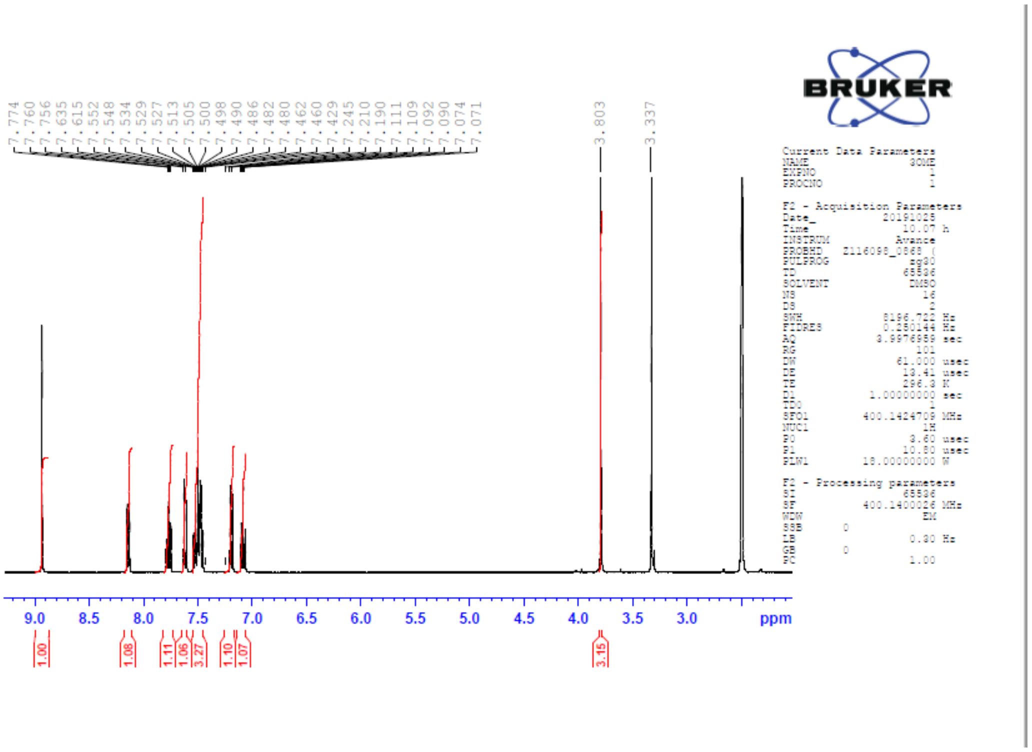

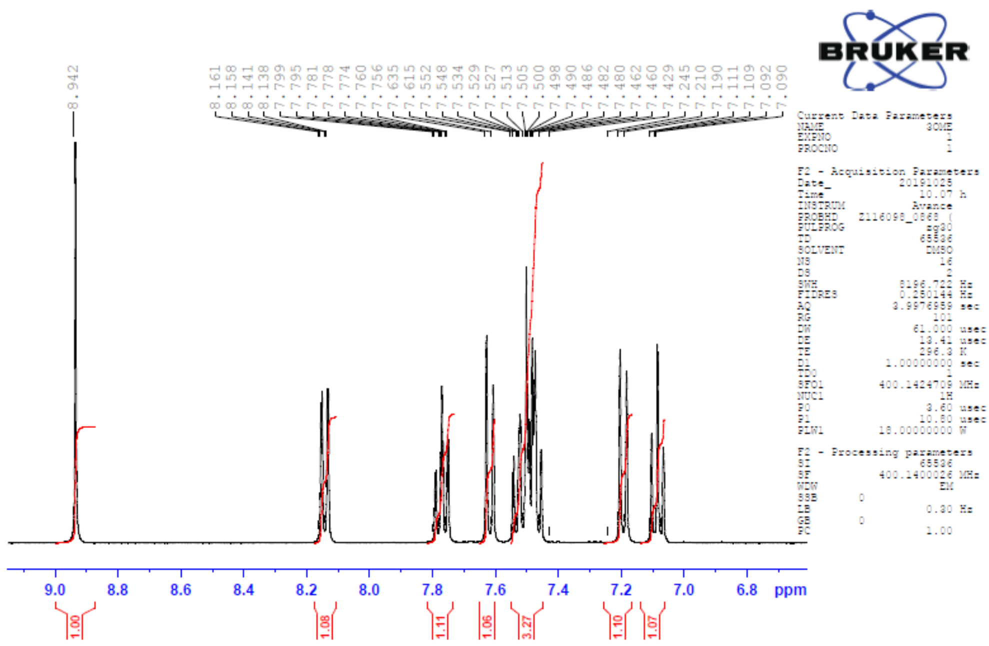
8.942
8.161
8.158
8.141
8.138
7.799
7.795
7.781
7.778
7.774
7.760
7.756
7.635
7.615
7.552
7.548
7.534
7.529
7.527
7.513
7.505
7.500
7.498
7.490
7.486
7.482
7.480
7.462
7.460
7.429
7.245
7.210
7.190
7.111
7.109
7.092
7.090
BRUKER
Current Data Parameters
NAME 3OME
EXPNO 1
PROCNO 1
F2 - Acquisition Parameters
Date_ 20191025
Time 10.07 h
INSTRUM Avance
PROBHD Z116098_0868 (
PULPROG zg30
TD 65536
SOLVENT DMSO
NS 16
DS 2
SWH 8196.722 Hz
FIDRES 0.250144 Hz
AQ 3.9976959 sec
RG 101
DW 61.000 usec
DE 13.41 usec
TE 296.3 K
D1 1.00000000 sec
TD0 1
SFO1 400.1424709 MHz
NUC1 1H
P0 3.60 usec
P1 10.80 usec
PLW1 18.00000000 W
F2 - Processing parameters
SI 65536
SF 400.1400026 MHz
WDW EM
SSB 0
LB 0.30 Hz
GB 0
PC 1.00
9.0
8.8
8.6
8.4
8.2
8.0
7.8
7.6
7.4
7.2
7.0
6.8
ppm
1.00
1.08
1.11
1.06
3.27
1.10
1.07

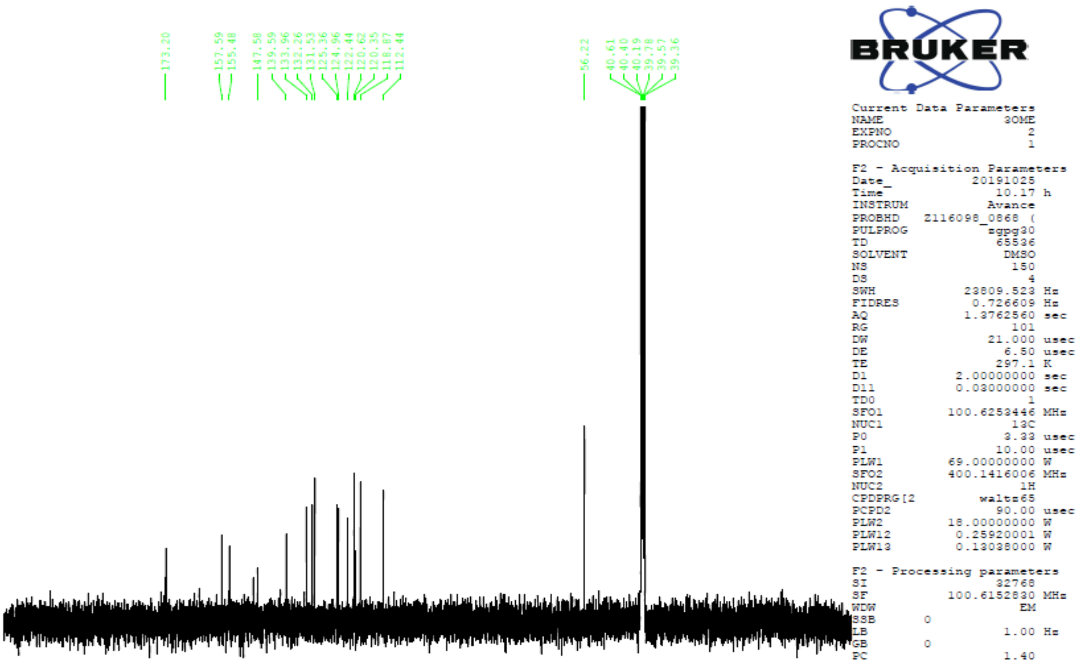
173.20
157.59
155.48
147.58
139.59
133.96
132.26
131.53
125.36
124.96
122.44
120.62
120.35
118.87
112.44
56.22
40.61
40.40
40.19
39.78
39.57
39.36
BRUKER
Current Data Parameters
NAME 3OME
EXPNO 2
PROCNO 1
F2 - Acquisition Parameters
Date_ 20191025
Time 10.17 h
INSTRUM Avance
PROBHD Z116098_0868 (
PULPROG zgpg30
TD 65536
SOLVENT DMSO
NS 150
DS 4
SWH 23809.523 Hz
FIDRES 0.726609 Hz
AQ 1.3762560 sec
RG 101
DW 21.000 usec
DE 6.50 usec
TE 297.1 K
D1 2.00000000 sec
D11 0.03000000 sec
TD0 1
SFO1 100.6253446 MHz
NUC1 13C
P0 3.33 usec
P1 10.00 usec
PLW1 69.00000000 W
SFO2 400.1416006 MHz
NUC2 1H
CPDPRG[2 waltz65
PCPD2 90.00 usec
PLW2 18.00000000 W
PLW12 0.25920001 W
PLW13 0.13038000 W
F2 - Processing parameters
SI 32768
SF 100.6152830 MHz
WDW EM
SSB 0
LB 1.00 Hz
GB 0
PC 1.40

3-hydroxy-2-o-tolyl-4H-chromen-4-one

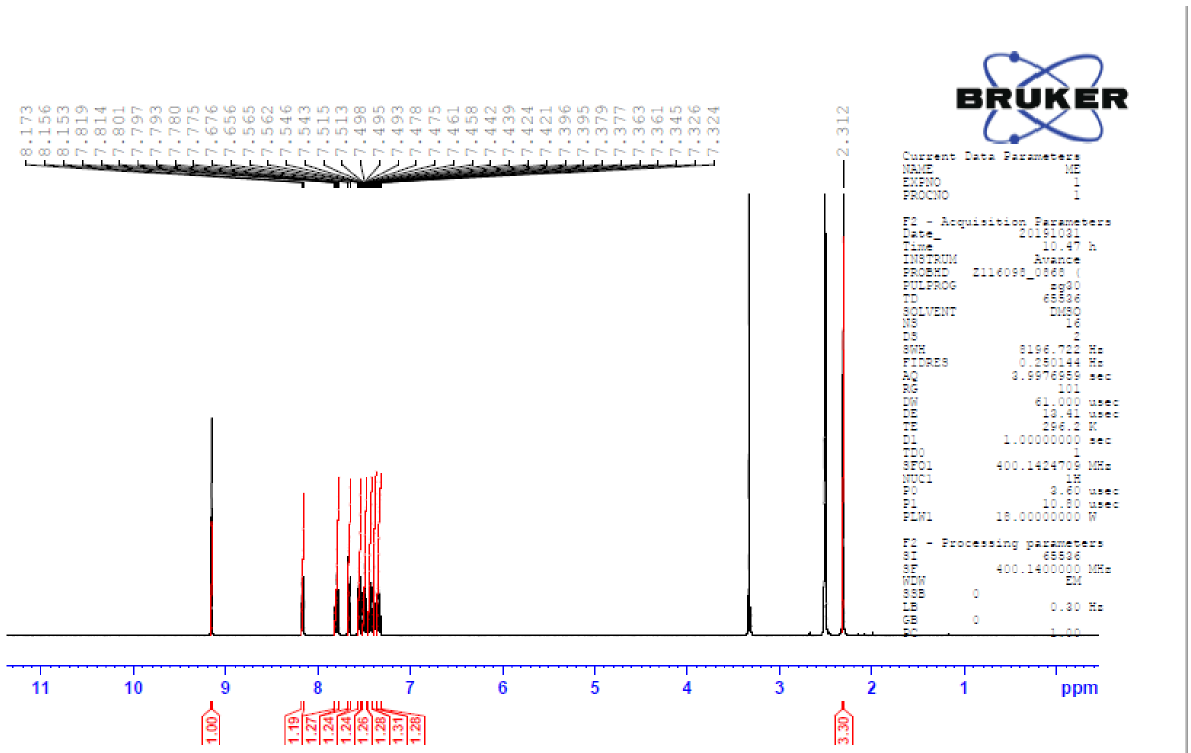

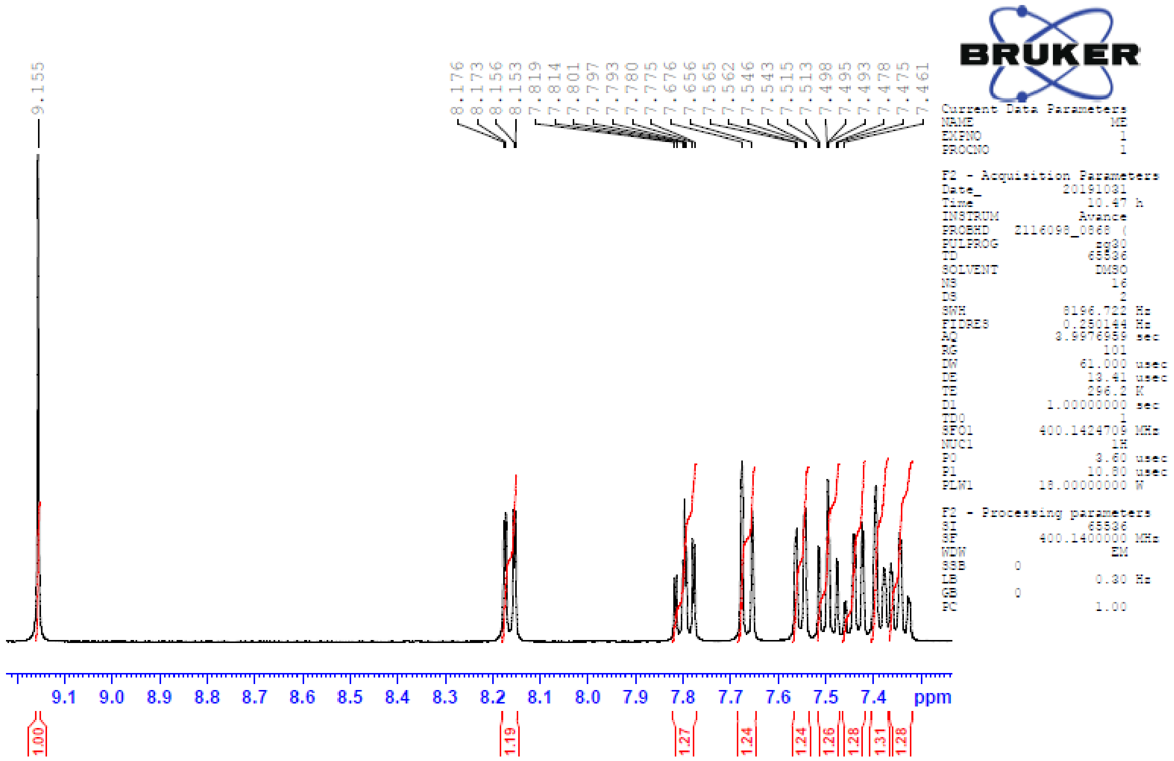

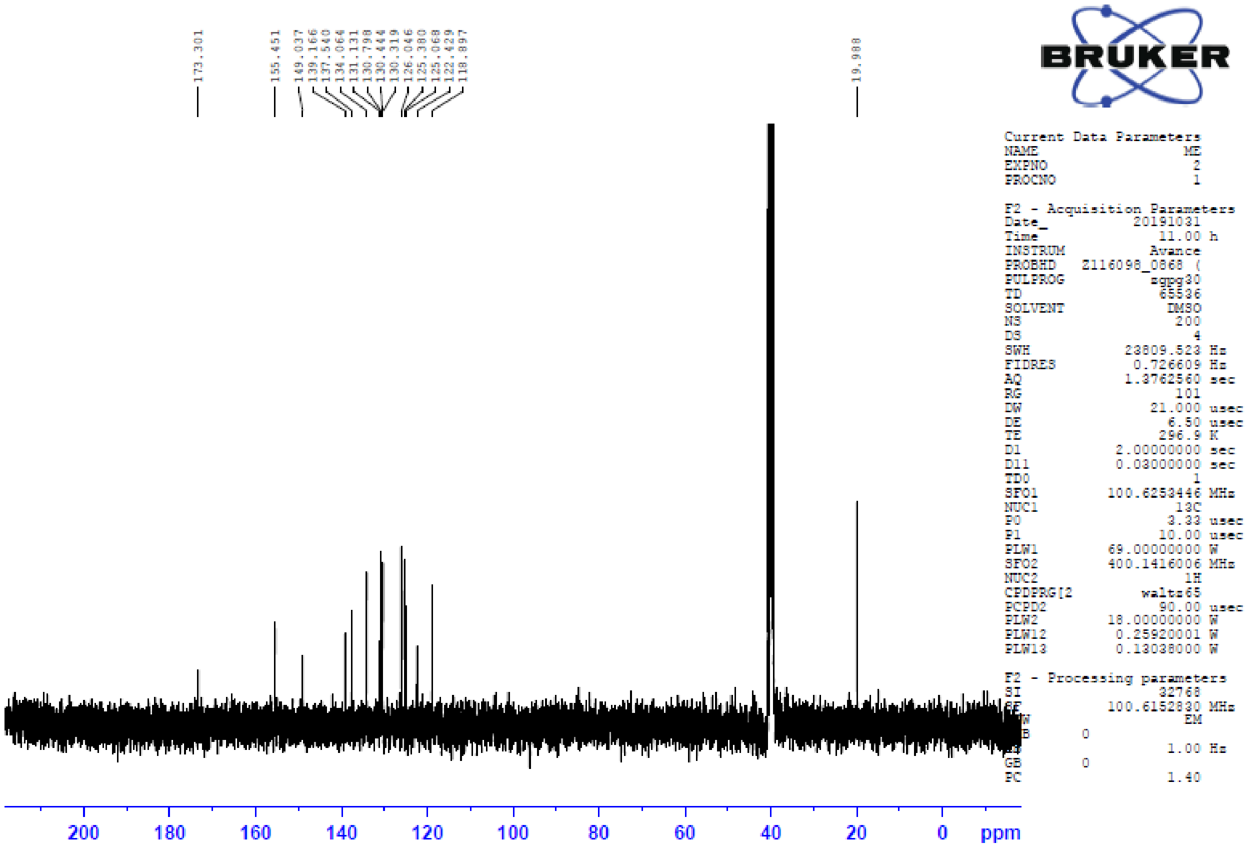

3. Discussion on the likelihood of proton transfer within the fourth singlet excited state

To understand the likelihood of proton transfer on the fourth singlet excited state, a relaxed scan was performed between the ground state optimized normal form and the tautomer form. This is performed by scanning on the distance between the hydroxy hydrogen and the carbonyl oxygen (from 2.06 to 0.92 Å) from the normal form to the tautomer form and the geometries were allowed to relax on the ground state using the same level of theory described in the main text. Vertical excitations were computed from these relaxed geometries and the corresponding profiles with the same excited state character were connected. (**Error! Reference source not found.**) It shows that there are intercrossing between the S3 and S4 states such that characterizing such a transition state for proton transfer would be difficult. However, if the molecules following the same character on the S4 state, it would require overcoming a barrier of ~ 8 kcal/mol (following the blue curve) when compared to a barrier of ~4 kcal/mol on the S1 state (following the orange curve). Nevertheless, both barriers are smaller than that on the ground state (~16 kcal/mol) This shows the excited states help to reduce the barrier for the proton transfer. However, the proton transfer within the S4 state is unlikely due to the barrier that it needs to overcome at room temperature. Given the potential conical intersections located on the profiles, it is more likely that the proton transfer occurs from the S4 state back to the lower excited states.

The authors are aware of the lack of the characterization of the transition states and cannot deny there might exist a lower barrier on the S4 state for the proton transfer. This energy profile is just providing an insight on the likelihood of the proton transfer within the same S4 excited state.

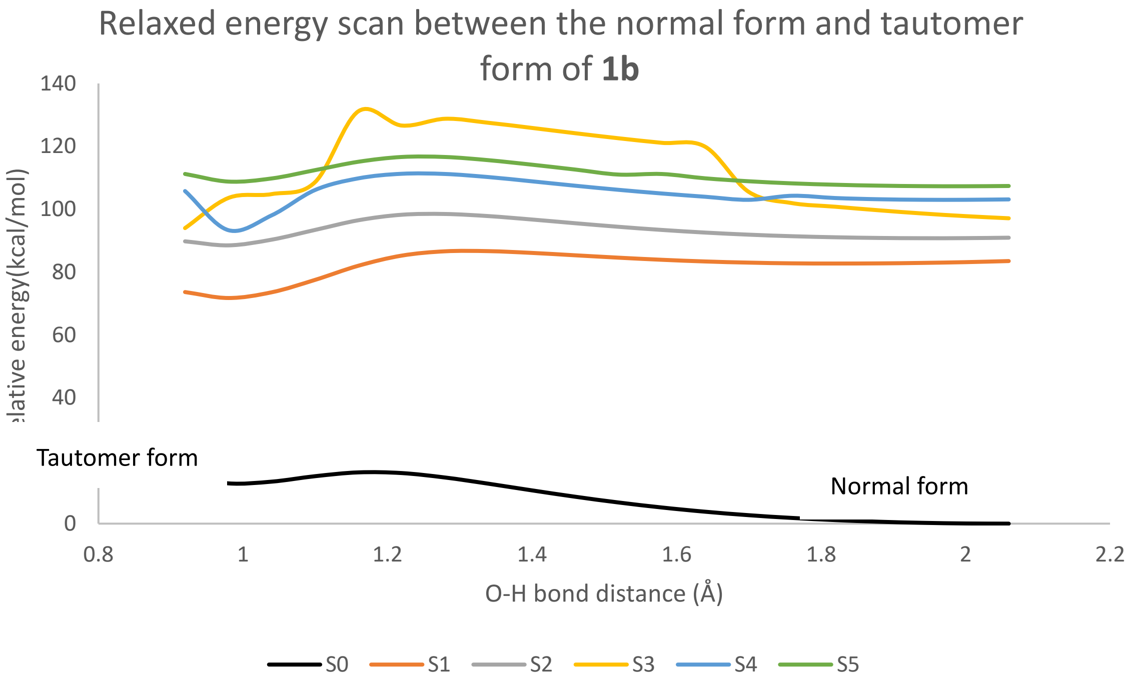

Figure S1. Relaxed energy scan from the proton transfer between the normal form and the tautomer form.

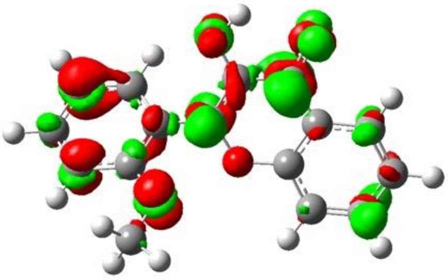

$\mathbf{S_2}$

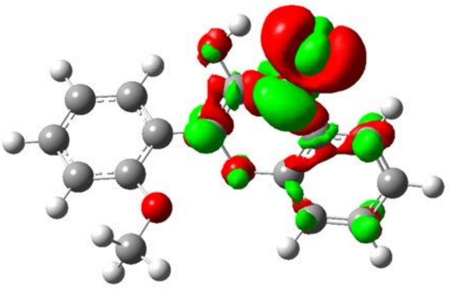

$\mathbf{S_3}$

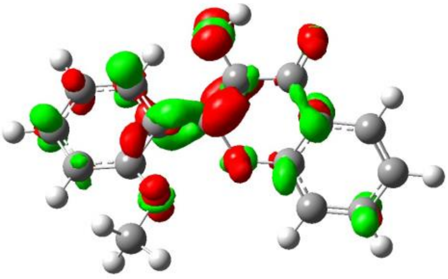

$\mathbf{S_5}$

Figure S2. The electron density difference plots for the S_2, S_3 and S_5 states for 1b as calculated relative to the S_0 ground state at the TD-B3LYP/6-311G(d) level of theory with SMD models in acetonitrile. The green contours depict the accumulation of electron density in the excited state, and the red contours illustrate the depletion of electron density from the S_0 ground state. The isocontour value is ±0.0024 au.

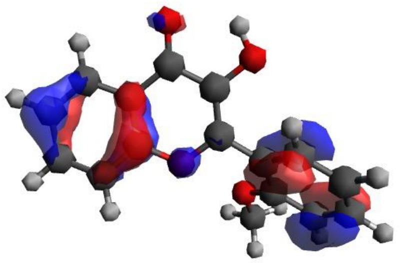

HOMO-2

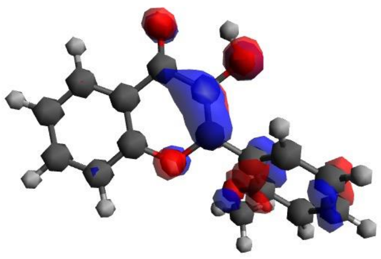

HOMO

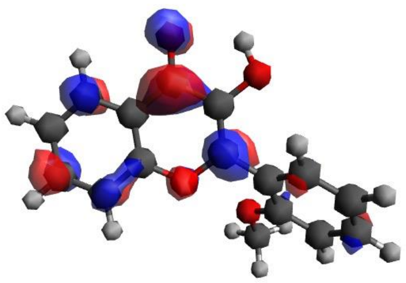

LUMO

Figure S3. Orbitals involved in the first and fourth lowest singlet state transitions.

4. Coordinates for Optimized Geometries (S_0, S_1, and S_4 of 1b)

Optimized S_0 geometry in scrf=(smd,solvent=acetonitrile) (rb3lyp= -918.040921 Hartree)

C 1.78499700 -0.71518200 -0.44562000
C 2.39160900 -1.90203500 -0.87324300
C 3.76870300 -2.01420000 -0.79944400
C 4.55267900 -0.95625400 -0.30276300
C 3.94731500 0.21139700 0.11779700
C 2.54744800 0.35223000 0.05228700
H 1.77335800 -2.70775100 -1.25208900
H 4.24644400 -2.93053900 -1.12908300
H 5.63068100 -1.06151500 -0.25155700
H 4.53086700 1.03900700 0.50478300
C 1.86606900 1.56415900 0.48556800
C 0.41490600 1.54257000 0.35963500
C -0.25053600 0.46328300 -0.13307300
C -1.70946800 0.36208400 -0.31025200
C -2.43875200 -0.71944300 0.23968500
C -2.39367100 1.34854600 -1.02783400
C -3.82017500 -0.79656300 0.03170400
C -3.76951600 1.27287900 -1.22767000
H -1.83154800 2.17888900 -1.43962500
C -4.47426300 0.19436800 -0.69947300
H -4.39143200 -1.61860900 0.44301500
H -4.28069800 2.04349300 -1.79400800
H -5.54638700 0.11716500 -0.84951000
O 2.41759500 2.57137000 0.95114100
O -0.22759300 2.65899400 0.78092800
H 0.47967000 3.25412600 1.09674200
O -1.73222600 -1.61410500 0.97112700
C -2.41962900 -2.71658000 1.56843100
H -2.88185500 -3.35481000 0.81029900
H -1.65609500 -3.28158600 2.09997700

H -3.17888700 -2.37482400 2.27729600
O 0.43217200 -0.64758300 -0.54452500

Excited State 1: Singlet-A 3.5970 eV 344.69 nm f=0.2858 <S**2>=0.000
69 -> 71 -0.20311
70 -> 71 0.66969
This state for optimization and/or second-order correction.
Total Energy, E(TD-HF/TD-DFT) = -917.908735214
Copying the excited state density for this state as the 1-particle RhoCI density.
Excited State 2: Singlet-A 3.9169 eV 316.54 nm f=0.0453 <S**2>=0.000
69 -> 71 0.66556
70 -> 71 0.19601
Excited State 3: Singlet-A 4.1832 eV 296.38 nm f=0.0017 <S**2>=0.000
65 -> 71 -0.16640
66 -> 71 0.43934
67 -> 71 0.46754
68 -> 71 0.20236
Excited State 4: Singlet-A 4.4419 eV 279.13 nm f=0.1108 <S**2>=0.000
66 -> 71 -0.12416
67 -> 71 -0.15329
68 -> 71 0.61574
70 -> 72 -0.22575
Excited State 5: Singlet-A 4.6254 eV 268.05 nm f=0.0323 <S**2>=0.000
65 -> 71 0.13649
66 -> 71 -0.16849
67 -> 71 0.10691
68 -> 71 0.19251
70 -> 72 0.60767
70 -> 73 0.10825
70 -> 74 0.10275

Optimized S_1 geometry in scrf=(smd,solvent=acetonitrile) (TD-DFT= -917.925676 Hartree)

C 1.77153500 -0.75226600 -0.24245900
C 2.31958900 -2.00699600 -0.43858600
C 3.71831100 -2.16571100 -0.39107600
C 4.53916000 -1.06673900 -0.14009600
C 3.98338300 0.19338700 0.06287500
C 2.58093400 0.37938500 0.01184500
H 1.66338300 -2.84858900 -0.62920800
H 4.14801300 -3.14865100 -0.54976000
H 5.61638000 -1.19266500 -0.10210200
H 4.60928700 1.05550100 0.26284900
C 1.94852800 1.64713800 0.21317000
C 0.48610400 1.64556700 0.16021400
C -0.29164100 0.49971100 -0.09959500
C -1.71394900 0.42618200 -0.14711300
C -2.45535600 -0.80803400 0.08248300
C -2.49115300 1.58911200 -0.43726800
C -3.85006100 -0.82567000 -0.01827800
C -3.86559200 1.54438100 -0.52065300
H -1.98115300 2.51988000 -0.63519700
C -4.55486900 0.33442300 -0.31633100
H -4.39093800 -1.74682400 0.15252500
H -4.41584500 2.44526000 -0.76964700

H -5.63610700 0.30045800 -0.39233000
O 2.50710400 2.75341000 0.46864600
O -0.04524300 2.82996100 0.43670500
H 0.75345800 3.40269500 0.58971200
O -1.74052600 -1.87763900 0.44462000
C -2.40360600 -3.10758900 0.77259600
H -2.94101300 -3.50108400 -0.09320500
H -1.60870900 -3.79312100 1.05633600
H -3.09141100 -2.96721100 1.60962800
O 0.39701000 -0.66614900 -0.32621800

Excited State 1: Singlet-A 2.8560 eV 434.11 nm f=0.7171 <S**2>=0.000
70 -> 71 -0.70135
This state for optimization and/or second-order correction.
Total Energy, E(TD-HF/TD-DFT) = -917.925675852
Copying the excited state density for this state as the 1-particle RhoCI density.
Excited State 2: Singlet-A 3.6436 eV 340.28 nm f=0.0068 <S**2>=0.000
69 -> 71 -0.69468
Excited State 3: Singlet-A 4.0662 eV 304.92 nm f=0.0914 <S**2>=0.000
66 -> 71 -0.39787
68 -> 71 0.48609
70 -> 72 -0.28952
Excited State 4: Singlet-A 4.0898 eV 303.16 nm f=0.0377 <S**2>=0.000
66 -> 71 0.45204
67 -> 71 -0.13205
70 -> 72 -0.50982
Excited State 5: Singlet-A 4.1540 eV 298.47 nm f=0.2284 <S**2>=0.000
66 -> 71 0.33029
68 -> 71 0.47905
70 -> 72 0.36399
Excited State 6: Singlet-A 4.5334 eV 273.49 nm f=0.1707 <S**2>=0.000
67 -> 71 -0.20421
70 -> 73 -0.66286
Excited State 7: Singlet-A 4.6066 eV 269.14 nm f=0.0677 <S**2>=0.000
67 -> 71 -0.61989
69 -> 72 -0.17882
70 -> 73 0.18662
70 -> 74 -0.11577
Excited State 8: Singlet-A 4.7775 eV 259.52 nm f=0.1428 <S**2>=0.000
67 -> 71 -0.18290
69 -> 72 0.56050
70 -> 74 0.33327
Excited State 9: Singlet-A 4.9259 eV 251.70 nm f=0.2203 <S**2>=0.000
65 -> 71 -0.27654
68 -> 72 0.14923
69 -> 72 0.32173
70 -> 74 -0.51211
Excited State 10: Singlet-A 5.1240 eV 241.97 nm f=0.0166 <S**2>=0.000
65 -> 71 0.58625
69 -> 72 0.16441
70 -> 74 -0.25687
70 -> 75 -0.19855
Excited State 11: Singlet-A 5.3066 eV 233.64 nm f=0.2183 <S**2>=0.000
67 -> 73 0.13484
68 -> 72 -0.60531

69 -> 74 -0.10270
70 -> 74 -0.12585
70 -> 75 0.23754
Excited State 12: Singlet-A 5.4639 eV 226.91 nm f=0.0176 <S**2>=0.000
65 -> 71 0.18112
67 -> 72 0.18979
68 -> 72 0.20926
69 -> 73 0.32144
70 -> 75 0.50165
Excited State 13: Singlet-A 5.5543 eV 223.22 nm f=0.1170 <S**2>=0.000
64 -> 71 0.13103
65 -> 71 0.12658
69 -> 73 -0.59419
70 -> 75 0.29064
Excited State 14: Singlet-A 5.6302 eV 220.21 nm f=0.0030 <S**2>=0.000
64 -> 71 0.26498
66 -> 72 0.63149
67 -> 72 -0.10365
Excited State 15: Singlet-A 5.6862 eV 218.05 nm f=0.0032 <S**2>=0.000
64 -> 71 -0.61953
66 -> 72 0.27774
Excited State 16: Singlet-A 5.8041 eV 213.61 nm f=0.1483 <S**2>=0.000
67 -> 72 -0.59043
68 -> 72 0.15503
68 -> 73 -0.22009
69 -> 74 -0.10197
70 -> 75 0.13919
Excited State 17: Singlet-A 5.8608 eV 211.55 nm f=0.3055 <S**2>=0.000
63 -> 71 0.10150
65 -> 72 0.11388
67 -> 72 0.10204
69 -> 74 -0.65149
Excited State 18: Singlet-A 6.1101 eV 202.92 nm f=0.0645 <S**2>=0.000
63 -> 71 -0.37592
65 -> 72 -0.24722
67 -> 72 0.19345
67 -> 73 0.21486
68 -> 72 0.11105
68 -> 73 -0.23010
68 -> 74 -0.24135
69 -> 75 0.26424
Excited State 19: Singlet-A 6.1450 eV 201.76 nm f=0.1043 <S**2>=0.000
63 -> 71 0.45078
65 -> 72 -0.20525
67 -> 72 0.18429
67 -> 73 0.17215
68 -> 73 -0.37349
68 -> 74 0.14555
Excited State 20: Singlet-A 6.1895 eV 200.31 nm f=0.3026 <S**2>=0.000
63 -> 71 -0.29507
65 -> 72 0.11319
67 -> 73 -0.16008
68 -> 73 -0.37520
68 -> 74 0.10833
69 -> 75 -0.44268

Excited State 21: Singlet-A 6.2883 eV 197.17 nm f=0.1559 <S**2>=0.000
62 -> 71 -0.11805
65 -> 72 0.19703
66 -> 73 0.27726
67 -> 73 -0.30925
68 -> 73 -0.19216
69 -> 74 0.10885
69 -> 75 0.27969
70 -> 76 -0.25487
70 -> 77 0.10054
70 -> 78 0.15828
Excited State 22: Singlet-A 6.3086 eV 196.53 nm f=0.0371 <S**2>=0.000
66 -> 73 0.28906
67 -> 73 -0.14122
69 -> 75 0.14217
70 -> 76 0.45984
70 -> 77 -0.20010
70 -> 78 -0.28866
70 -> 79 -0.12901
Excited State 23: Singlet-A 6.3408 eV 195.53 nm f=0.0498 <S**2>=0.000
62 -> 71 0.15118
65 -> 72 -0.14719
66 -> 73 0.55312
67 -> 73 0.15769
68 -> 73 0.17590
69 -> 75 -0.17986
70 -> 76 -0.12794
Excited State 24: Singlet-A 6.4550 eV 192.07 nm f=0.0123 <S**2>=0.000
60 -> 71 0.22832
61 -> 71 -0.40510
62 -> 71 -0.12112
64 -> 72 0.13753
65 -> 72 0.19433
67 -> 73 0.15347
68 -> 74 -0.35711
69 -> 75 -0.16743
Excited State 25: Singlet-A 6.4835 eV 191.23 nm f=0.3369 <S**2>=0.000
61 -> 71 0.29285
62 -> 71 -0.26504
65 -> 72 0.38091
67 -> 73 0.32372
68 -> 74 0.12165
68 -> 75 0.20140
Excited State 26: Singlet-A 6.5043 eV 190.62 nm f=0.2698 <S**2>=0.000
60 -> 71 0.10196
61 -> 71 -0.38218
63 -> 71 -0.17552
68 -> 74 0.47939
69 -> 75 0.19132
Excited State 27: Singlet-A 6.5186 eV 190.20 nm f=0.0037 <S**2>=0.000
70 -> 76 -0.43232
70 -> 77 -0.25464
70 -> 78 -0.46656
Excited State 28: Singlet-A 6.5605 eV 188.99 nm f=0.0477 <S**2>=0.000
60 -> 71 -0.35568

61 -> 71 -0.10004
62 -> 71 0.44703
65 -> 72 0.27301
67 -> 73 0.10900
Excited State 29: Singlet-A 6.5777 eV 188.49 nm f=0.0065 <S**2>=0.000
69 -> 76 -0.11829
70 -> 77 -0.59599
70 -> 78 0.29686
70 -> 79 0.15321
Excited State 30: Singlet-A 6.7636 eV 183.31 nm f=0.0370 <S**2>=0.000
59 -> 71 -0.18390
60 -> 71 0.44229
61 -> 71 0.19481
62 -> 71 0.33984
67 -> 74 -0.17253
67 -> 75 0.11059
68 -> 75 -0.13993
Excited State 31: Singlet-A 6.8329 eV 181.45 nm f=0.1821 <S**2>=0.000
60 -> 71 -0.13177
67 -> 73 0.20037
68 -> 75 -0.60837
Excited State 32: Singlet-A 6.8634 eV 180.64 nm f=0.0023 <S**2>=0.000
59 -> 71 0.52616
66 -> 74 0.19098
66 -> 75 -0.13897
67 -> 74 -0.33091
Excited State 33: Singlet-A 6.8855 eV 180.07 nm f=0.0315 <S**2>=0.000
59 -> 71 0.32913
60 -> 71 0.16447
62 -> 71 0.11430
65 -> 73 0.14219
67 -> 74 0.53398
Excited State 34: Singlet-A 6.9240 eV 179.06 nm f=0.0108 <S**2>=0.000
59 -> 71 -0.14017
66 -> 74 0.58652
66 -> 75 -0.24264
67 -> 74 0.21314
Excited State 35: Singlet-A 6.9765 eV 177.72 nm f=0.0062 <S**2>=0.000
60 -> 71 0.11848
64 -> 72 -0.50409
70 -> 78 0.14376
70 -> 79 -0.37618
Excited State 36: Singlet-A 6.9790 eV 177.65 nm f=0.0037 <S**2>=0.000
64 -> 72 0.38677
69 -> 77 -0.11743
70 -> 78 0.17657
70 -> 79 -0.50037
Excited State 37: Singlet-A 7.0091 eV 176.89 nm f=0.1119 <S**2>=0.000
65 -> 73 -0.62334
67 -> 74 0.13891
67 -> 75 0.22561
Excited State 38: Singlet-A 7.1020 eV 174.58 nm f=0.0378 <S**2>=0.000
66 -> 74 -0.22521
66 -> 75 -0.45312
67 -> 75 0.12417

69 -> 76 0.15044
70 -> 80 -0.36830
Excited State 39: Singlet-A 7.1160 eV 174.23 nm f=0.0217 <S**2>=0.000
65 -> 73 0.10704
66 -> 74 -0.17523
66 -> 75 -0.34975
69 -> 76 -0.24287
69 -> 77 0.15292
70 -> 80 0.41656
70 -> 81 0.16091
Excited State 40: Singlet-A 7.1831 eV 172.60 nm f=0.0400 <S**2>=0.000
65 -> 74 -0.27428
68 -> 77 -0.10726
69 -> 76 -0.46104
69 -> 77 0.22143
70 -> 80 -0.30846
Excited State 41: Singlet-A 7.2019 eV 172.15 nm f=0.1311 <S**2>=0.000
63 -> 72 -0.18406
65 -> 74 0.56588
68 -> 75 0.10267
69 -> 76 -0.24241
69 -> 77 0.10998
70 -> 80 -0.11591
Excited State 42: Singlet-A 7.3034 eV 169.76 nm f=0.1431 <S**2>=0.000
56 -> 71 0.11566
65 -> 73 -0.15944
66 -> 75 -0.15155
67 -> 75 -0.57532
70 -> 81 -0.20670
Excited State 43: Singlet-A 7.3277 eV 169.20 nm f=0.0049 <S**2>=0.000
56 -> 71 0.34451
57 -> 71 0.27917
61 -> 72 0.12417
68 -> 76 0.15159
69 -> 76 0.21112
69 -> 77 0.38147
70 -> 79 -0.10216
Excited State 44: Singlet-A 7.3379 eV 168.96 nm f=0.0129 <S**2>=0.000
56 -> 71 0.24104
57 -> 71 0.35793
58 -> 71 -0.23497
67 -> 75 0.11643
68 -> 76 -0.13168
69 -> 76 -0.18738
69 -> 77 -0.33189
Excited State 45: Singlet-A 7.3598 eV 168.46 nm f=0.0736 <S**2>=0.000
56 -> 71 0.23929
58 -> 71 0.26510
63 -> 72 -0.32447
69 -> 77 -0.18892
69 -> 78 -0.10092
70 -> 81 0.37946
Excited State 46: Singlet-A 7.3988 eV 167.57 nm f=0.0057 <S**2>=0.000
56 -> 71 0.26462
57 -> 71 -0.15947

58 -> 71 0.29323
60 -> 72 -0.10039
67 -> 75 0.18311
70 -> 80 0.15402
70 -> 81 -0.41259
Excited State 47: Singlet-A 7.4314 eV 166.84 nm f=0.0409 <S**2>=0.000
58 -> 71 0.26450
63 -> 72 0.52547
65 -> 74 0.17189
69 -> 78 -0.22432
70 -> 81 0.15915
Excited State 48: Singlet-A 7.4591 eV 166.22 nm f=0.0123 <S**2>=0.000
58 -> 71 -0.14764
63 -> 72 -0.16307
68 -> 76 0.20070
69 -> 78 -0.56137
70 -> 82 -0.18598
Excited State 49: Singlet-A 7.4838 eV 165.67 nm f=0.0438 <S**2>=0.000
56 -> 71 -0.24205
57 -> 71 0.42524
58 -> 71 0.38915
63 -> 72 -0.14124
64 -> 74 -0.12333
Excited State 50: Singlet-A 7.5868 eV 163.42 nm f=0.0035 <S**2>=0.000
61 -> 72 -0.20255
61 -> 73 0.17967
64 -> 73 0.50215
70 -> 82 0.29315

S_4 geometry in scrf=(smd,solvent=acetonitrile) (TD-DFT= -917.925676 Hartree)
C 1.78244800 -0.74254300 -0.36528900
C 2.38215600 -1.94120700 -0.72596900
C 3.77718900 -2.04928900 -0.70048200
C 4.56001800 -0.96090900 -0.31439200
C 3.96420500 0.24433000 0.04572000
C 2.55825100 0.37221600 0.02398300
H 1.75421400 -2.77192000 -1.02758300
H 4.24424500 -2.98601400 -0.98359600
H 5.64088000 -1.05024100 -0.29219100
H 4.57504000 1.08874900 0.34243000
C 1.85530900 1.56053600 0.40172500
C 0.44818100 1.58955300 0.33068700
C -0.26053100 0.47371100 -0.08877600
C -1.70760600 0.39021300 -0.24896700
C -2.44285200 -0.74745500 0.19094900
C -2.41846100 1.44304400 -0.85290000
C -3.82406800 -0.80634500 -0.01543600
C -3.79484700 1.38454700 -1.03920200
H -1.86882600 2.31196900 -1.19206100
C -4.49544200 0.25284100 -0.62718200
H -4.38683700 -1.66970200 0.31543400
H -4.31152700 2.20987200 -1.51738900
H -5.56859900 0.18661100 -0.77409200
O 2.53063700 2.59935000 0.85932400
O -0.24555500 2.68796900 0.75890000

H 0.25529800 3.49636200 0.58179300
O -1.74274600 -1.71285300 0.83662900
C -2.43268200 -2.87283000 1.30320200
H -2.88912600 -3.42519000 0.47687500
H -1.67298400 -3.49332300 1.77560100
H -3.19847100 -2.61374600 2.04005600
O 0.42255000 -0.67770400 -0.41126600

Excited State 1: Singlet-A 3.2822 eV 377.74 nm f=0.5447 <S**2>=0.000
69 -> 71 0.19062
70 -> 71 -0.67166
Excited State 2: Singlet-A 4.1170 eV 301.15 nm f=0.3181 <S**2>=0.000
67 -> 71 -0.67379
68 -> 71 0.12961
Excited State 3: Singlet-A 3.6864 eV 336.33 nm f=0.0569 <S**2>=0.000
68 -> 71 0.15121
69 -> 71 -0.65672
70 -> 71 -0.16829
Excited State 4: Singlet-A 3.4037 eV 364.27 nm f=0.0039 <S**2>=0.000
67 -> 71 0.13601
68 -> 71 0.66508
69 -> 71 0.13688
This state for optimization and/or second-order correction.
Total Energy, E(TD-HF/TD-DFT) = -917.899862823
Copying the excited state density for this state as the 1-particle RhoCI density.
Excited State 5: Singlet-A 4.4393 eV 279.29 nm f=0.0034 <S**2>=0.000
66 -> 71 -0.45672
70 -> 72 0.51320
Excited State 6: Singlet-A 4.4927 eV 275.97 nm f=0.1544 <S**2>=0.000
66 -> 71 -0.51177
70 -> 72 -0.45165
Excited State 7: Singlet-A 4.7653 eV 260.18 nm f=0.1785 <S**2>=0.000
69 -> 72 0.66251
70 -> 73 -0.13322
70 -> 74 0.11340
Excited State 8: Singlet-A 4.9754 eV 249.19 nm f=0.0331 <S**2>=0.000
65 -> 71 -0.54153
68 -> 72 0.10287
69 -> 73 -0.12976
70 -> 72 -0.11479
70 -> 73 -0.29108
70 -> 74 -0.19693
Excited State 9: Singlet-A 4.9870 eV 248.62 nm f=0.0792 <S**2>=0.000
65 -> 71 -0.27279
68 -> 72 0.11239
69 -> 72 0.10149
70 -> 73 0.60725
Excited State 10: Singlet-A 5.0406 eV 245.97 nm f=0.0036 <S**2>=0.000
65 -> 71 -0.14376
67 -> 72 -0.11422
68 -> 72 -0.66309
Excited State 11: Singlet-A 5.2376 eV 236.72 nm f=0.0946 <S**2>=0.000
65 -> 71 0.22680
67 -> 72 -0.37271
69 -> 72 0.10737

69 -> 73 -0.13688
69 -> 75 -0.13924
70 -> 74 -0.44893
70 -> 75 -0.13002
Excited State 12: Singlet-A 5.3525 eV 231.64 nm f=0.1057 <S**2>=0.000
66 -> 73 -0.13507
67 -> 72 0.46317
68 -> 72 -0.10545
69 -> 72 0.10566
69 -> 74 -0.14559
70 -> 74 -0.42048
70 -> 75 0.11064
Excited State 13: Singlet-A 5.4694 eV 226.69 nm f=0.0063 <S**2>=0.000
66 -> 72 0.29852
69 -> 73 -0.56172
70 -> 74 0.11053
70 -> 75 -0.19399
Excited State 14: Singlet-A 5.5829 eV 222.08 nm f=0.1249 <S**2>=0.000
64 -> 71 0.15925
65 -> 71 0.12461
66 -> 72 -0.22739
67 -> 73 -0.10650
69 -> 73 -0.28996
70 -> 74 0.10338
70 -> 75 0.51454
Excited State 15: Singlet-A 5.6698 eV 218.67 nm f=0.2487 <S**2>=0.000
64 -> 71 0.11102
66 -> 72 0.48253
67 -> 72 -0.27339
67 -> 73 0.11402
68 -> 73 -0.10081
69 -> 73 0.14585
70 -> 75 0.29340
Excited State 16: Singlet-A 5.7038 eV 217.37 nm f=0.0232 <S**2>=0.000
67 -> 73 -0.10790
68 -> 73 -0.60253
68 -> 74 -0.11625
68 -> 75 0.12217
69 -> 74 -0.23993
Excited State 17: Singlet-A 5.7569 eV 215.37 nm f=0.2110 <S**2>=0.000
63 -> 71 -0.10763
64 -> 71 -0.15333
68 -> 73 -0.24277
69 -> 73 0.11061
69 -> 74 0.56412
69 -> 75 -0.12100
Excited State 18: Singlet-A 5.8575 eV 211.67 nm f=0.1048 <S**2>=0.000
61 -> 71 -0.13422
63 -> 71 -0.14860
64 -> 71 -0.58150
69 -> 74 -0.21167
70 -> 75 0.15530
Excited State 19: Singlet-A 5.9366 eV 208.85 nm f=0.0692 <S**2>=0.000
64 -> 71 0.12263
65 -> 72 0.21260

66 -> 72 0.11629
66 -> 73 0.16571
67 -> 74 -0.10728
69 -> 74 -0.10747
69 -> 75 -0.57065
Excited State 20: Singlet-A 6.0676 eV 204.34 nm f=0.2546 <S**2>=0.000
63 -> 71 -0.14386
65 -> 72 0.29029
66 -> 72 0.22183
66 -> 73 0.21210
67 -> 73 -0.48515
68 -> 73 0.11272
69 -> 75 0.16592
Excited State 21: Singlet-A 6.0965 eV 203.37 nm f=0.1564 <S**2>=0.000
63 -> 71 0.59373
64 -> 71 -0.18023
65 -> 72 0.11721
67 -> 74 -0.12226
68 -> 74 0.16265
69 -> 75 0.11819
Excited State 22: Singlet-A 6.1218 eV 202.53 nm f=0.0024 <S**2>=0.000
63 -> 71 -0.12625
67 -> 74 0.14398
68 -> 73 -0.16309
68 -> 74 0.45808
68 -> 75 -0.44175
Excited State 23: Singlet-A 6.2341 eV 198.88 nm f=0.0644 <S**2>=0.000
62 -> 71 -0.47009
63 -> 71 -0.15014
65 -> 72 0.10592
66 -> 73 0.32680
67 -> 73 0.26725
Excited State 24: Singlet-A 6.2783 eV 197.48 nm f=0.0571 <S**2>=0.000
62 -> 71 -0.37025
65 -> 72 0.17236
66 -> 73 -0.30293
67 -> 73 -0.15633
67 -> 74 0.32465
67 -> 75 -0.19681
69 -> 75 -0.16799
Excited State 25: Singlet-A 6.3408 eV 195.54 nm f=0.0706 <S**2>=0.000
61 -> 71 -0.14094
68 -> 74 -0.46994
68 -> 75 -0.45786
Excited State 26: Singlet-A 6.3740 eV 194.52 nm f=0.0724 <S**2>=0.000
61 -> 71 0.24815
62 -> 71 -0.13949
65 -> 72 -0.34482
67 -> 73 -0.21386
67 -> 75 0.14159
68 -> 75 -0.16325
70 -> 76 -0.36872
Excited State 27: Singlet-A 6.3819 eV 194.28 nm f=0.1432 <S**2>=0.000
65 -> 72 -0.27134
66 -> 73 0.10821

67 -> 73 -0.12276
67 -> 74 0.20189
67 -> 75 0.21407
69 -> 75 -0.10690
70 -> 76 0.48901
Excited State 28: Singlet-A 6.3853 eV 194.17 nm f=0.2522 <S**2>=0.000
61 -> 71 0.35296
62 -> 71 -0.10557
63 -> 71 -0.10524
65 -> 72 0.10879
66 -> 73 -0.19809
67 -> 74 -0.41361
67 -> 75 -0.12323
68 -> 75 -0.13872
70 -> 76 0.25027
Excited State 29: Singlet-A 6.4615 eV 191.88 nm f=0.0563 <S**2>=0.000
60 -> 71 0.14987
61 -> 71 0.46365
62 -> 71 0.21785
64 -> 71 -0.13380
65 -> 72 0.10992
66 -> 73 0.10144
66 -> 75 -0.13221
67 -> 73 0.10074
67 -> 74 0.29839
Excited State 30: Singlet-A 6.5614 eV 188.96 nm f=0.2360 <S**2>=0.000
60 -> 71 0.10999
65 -> 72 -0.16704
66 -> 73 0.28463
66 -> 74 -0.25936
67 -> 75 -0.50237
Excited State 31: Singlet-A 6.6543 eV 186.32 nm f=0.2519 <S**2>=0.000
65 -> 72 0.15060
66 -> 74 -0.61278
67 -> 75 0.22979
Excited State 32: Singlet-A 6.8031 eV 182.25 nm f=0.0529 <S**2>=0.000
59 -> 71 -0.16898
60 -> 71 -0.35602
65 -> 73 -0.12456
66 -> 74 -0.17280
66 -> 75 -0.50996
Excited State 33: Singlet-A 6.8494 eV 181.02 nm f=0.0343 <S**2>=0.000
59 -> 71 0.22814
60 -> 71 0.35755
62 -> 71 -0.10010
65 -> 73 -0.43274
66 -> 75 -0.21265
69 -> 76 0.11710
Excited State 34: Singlet-A 6.8558 eV 180.85 nm f=0.0115 <S**2>=0.000
65 -> 73 -0.11029
69 -> 76 -0.42076
69 -> 78 0.15820
70 -> 76 0.16666
70 -> 77 -0.26289
70 -> 78 -0.38422

Excited State 35: Singlet-A 6.9266 eV 179.00 nm f=0.0103 <S**2>=0.000
59 -> 71 0.56216
60 -> 71 -0.21952
64 -> 72 0.16782
65 -> 73 0.20278
Excited State 36: Singlet-A 6.9732 eV 177.80 nm f=0.2492 <S**2>=0.000
59 -> 71 0.13690
60 -> 71 -0.29812
65 -> 73 -0.44226
65 -> 74 0.16183
66 -> 75 0.29307
68 -> 76 -0.12934
Excited State 37: Singlet-A 7.0188 eV 176.65 nm f=0.1524 <S**2>=0.000
64 -> 72 0.22125
65 -> 74 0.58431
70 -> 77 -0.16050
Excited State 38: Singlet-A 7.0214 eV 176.58 nm f=0.0362 <S**2>=0.000
65 -> 74 -0.13624
69 -> 76 0.25521
70 -> 77 -0.58241
70 -> 78 0.12107
70 -> 79 -0.10111
Excited State 39: Singlet-A 7.0920 eV 174.82 nm f=0.0211 <S**2>=0.000
59 -> 71 0.10938
63 -> 72 -0.13748
64 -> 72 -0.37486
69 -> 76 -0.31250
69 -> 77 -0.12880
70 -> 78 0.39604
Excited State 40: Singlet-A 7.1043 eV 174.52 nm f=0.0182 <S**2>=0.000
59 -> 71 -0.13089
63 -> 72 0.17550
64 -> 72 0.39651
65 -> 74 -0.19396
69 -> 76 -0.28234
69 -> 77 -0.12232
70 -> 78 0.32467
Excited State 41: Singlet-A 7.2082 eV 172.00 nm f=0.0760 <S**2>=0.000
65 -> 75 0.12637
68 -> 76 0.58950
68 -> 78 0.15682
69 -> 78 0.11730
70 -> 80 -0.11447
Excited State 42: Singlet-A 7.2590 eV 170.80 nm f=0.0183 <S**2>=0.000
56 -> 71 0.20354
57 -> 71 -0.16171
58 -> 71 0.41906
63 -> 72 -0.14308
64 -> 72 0.14337
65 -> 75 0.31038
69 -> 77 -0.16674
70 -> 79 0.13003
Excited State 43: Singlet-A 7.2638 eV 170.69 nm f=0.0221 <S**2>=0.000
58 -> 71 -0.18379
67 -> 78 -0.10389

69 -> 77 -0.43324
69 -> 78 -0.10943
70 -> 79 0.40301
Excited State 44: Singlet-A 7.3046 eV 169.73 nm f=0.1055 <S**2>=0.000
56 -> 71 -0.18271
57 -> 71 0.15102
58 -> 71 -0.27990
63 -> 72 -0.17230
65 -> 74 -0.10868
65 -> 75 0.51607
Excited State 45: Singlet-A 7.3561 eV 168.55 nm f=0.0026 <S**2>=0.000
63 -> 72 -0.15347
67 -> 76 -0.19916
69 -> 76 0.13898
69 -> 78 0.50654
69 -> 79 -0.12819
70 -> 79 0.26698
Excited State 46: Singlet-A 7.3692 eV 168.25 nm f=0.1980 <S**2>=0.000
63 -> 72 -0.55849
64 -> 72 0.19434
65 -> 75 -0.27066
Excited State 47: Singlet-A 7.4405 eV 166.63 nm f=0.0044 <S**2>=0.000
56 -> 71 0.21034
57 -> 71 0.30230
61 -> 72 -0.22969
62 -> 72 0.45048
64 -> 73 0.16991
Excited State 48: Singlet-A 7.4720 eV 165.93 nm f=0.0026 <S**2>=0.000
67 -> 76 0.17222
67 -> 77 -0.10598
69 -> 77 0.42583
69 -> 78 -0.17446
70 -> 78 0.16510
70 -> 79 0.41655
Excited State 49: Singlet-A 7.4814 eV 165.72 nm f=0.0275 <S**2>=0.000
54 -> 71 0.19753
55 -> 71 0.14143
56 -> 71 0.12333
57 -> 71 -0.43637
58 -> 71 -0.32340
61 -> 72 -0.17475
70 -> 80 0.18115
Excited State 50: Singlet-A 7.5295 eV 164.66 nm f=0.0073 <S**2>=0.000
56 -> 71 -0.32843
57 -> 71 -0.25991
62 -> 72 0.38381
63 -> 72 0.12027
70 -> 80 -0.32167

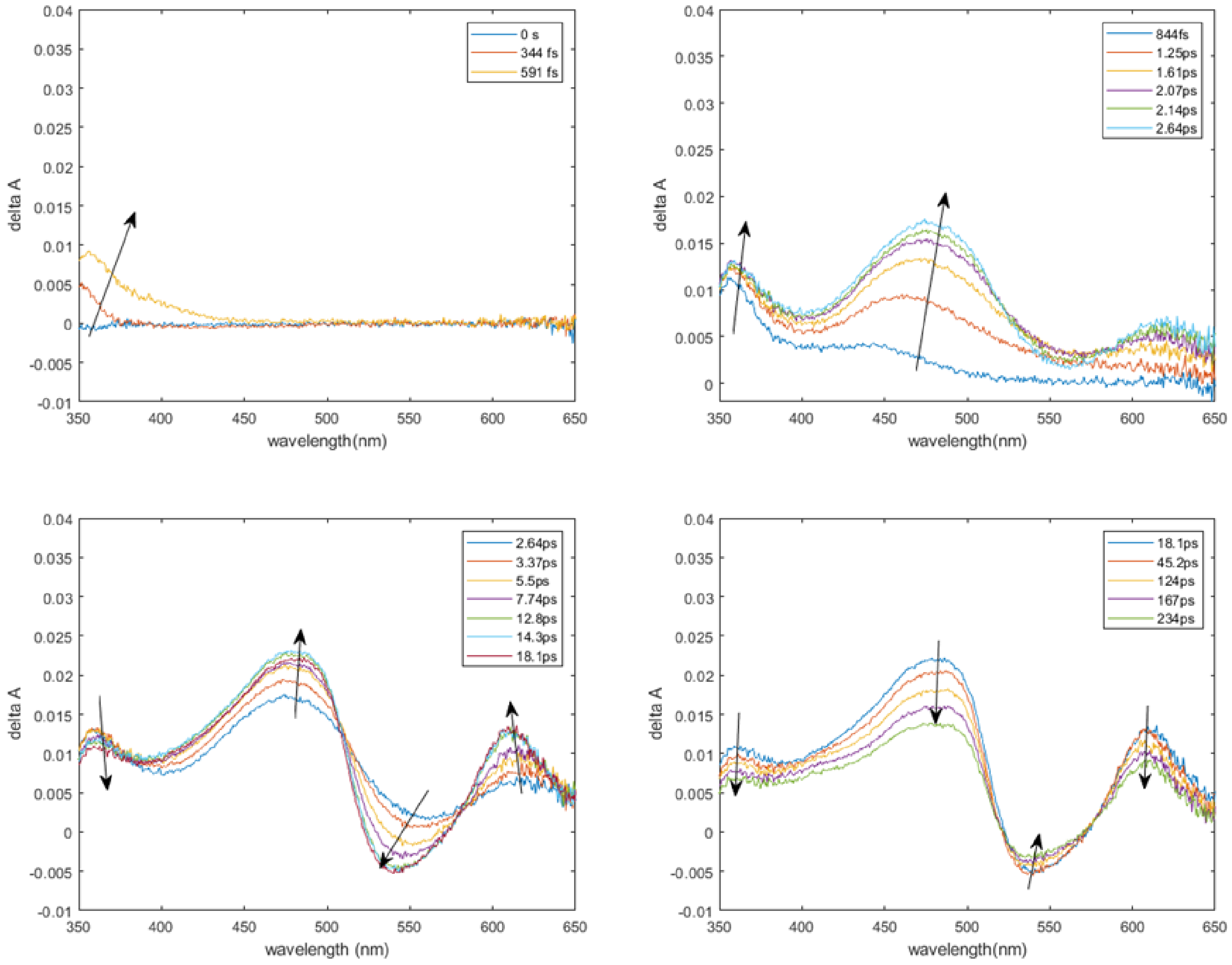

Figure S4. Transient UV-vis absorption spectra produced by fs laser excitation of **1c** in acetonitrile. (λ_{max} = 266 nm) with different time frames (**a**) <1 ps; (**b**) 1–2 ps; (**c**) 2–18 ps and (**d**) 18–300 ps. The arrows indicate the growth and decay of peaks.

Table S1. Vertical excitation energies, oscillator strengths, and the dominant occupied and virtual orbitals contributing to the four lowest energy singlet excitations of **1c** at the level of theory of TD-B3LYP/6-311G(d) with universal solvation model (SMD) model in acetonitrile.

Excited State	Energy (eV) (Wavelength (nm))	Major Character (% Contributions)	Oscillator Strength
S_1	3.70 (335)	HOMO → LUMO (96%)	0.3298
S_2	4.15 (299)	HOMO-4→LUMO (40%) HOMO-3→LUMO (32%)	0.008
S_3	4.29 (289)	HOMO-1→LUMO (81%)	0.0382
S_4	4.40 (281)	HOMO-2→LUMO (83%)	0.0928
S_5	4.66 (266)	HOMO→LUMO + 1 (74%)	0.0323

Table S2. Transient kinetics observed at various probe wavelengths for **1c.**

	Growth Lifetime	Decay Lifetime

**Transient Absorptions (nm)**	**τ_r, ps (A_r)**	**τ_1, ps (A_1)<br>τ_2, ps (A_2)**
362	<0.1	16.5 ± 0.3 (2.2×10^{-3})<br>423 ± 16 (8.0×10^{-3})
481	1.7 ± 0.1 (2.6×10^{-2})	417 ± 5.1 (2.1×10^{-2})
540	4.2 ± 0.1 (-1.0×10^{-2})	393 ± 8.4 (-6.5×10^{-3})
616	4.2 ± 0.2 (1.4×10^{-2})	397 ± 13 (1.2×10^{-2})

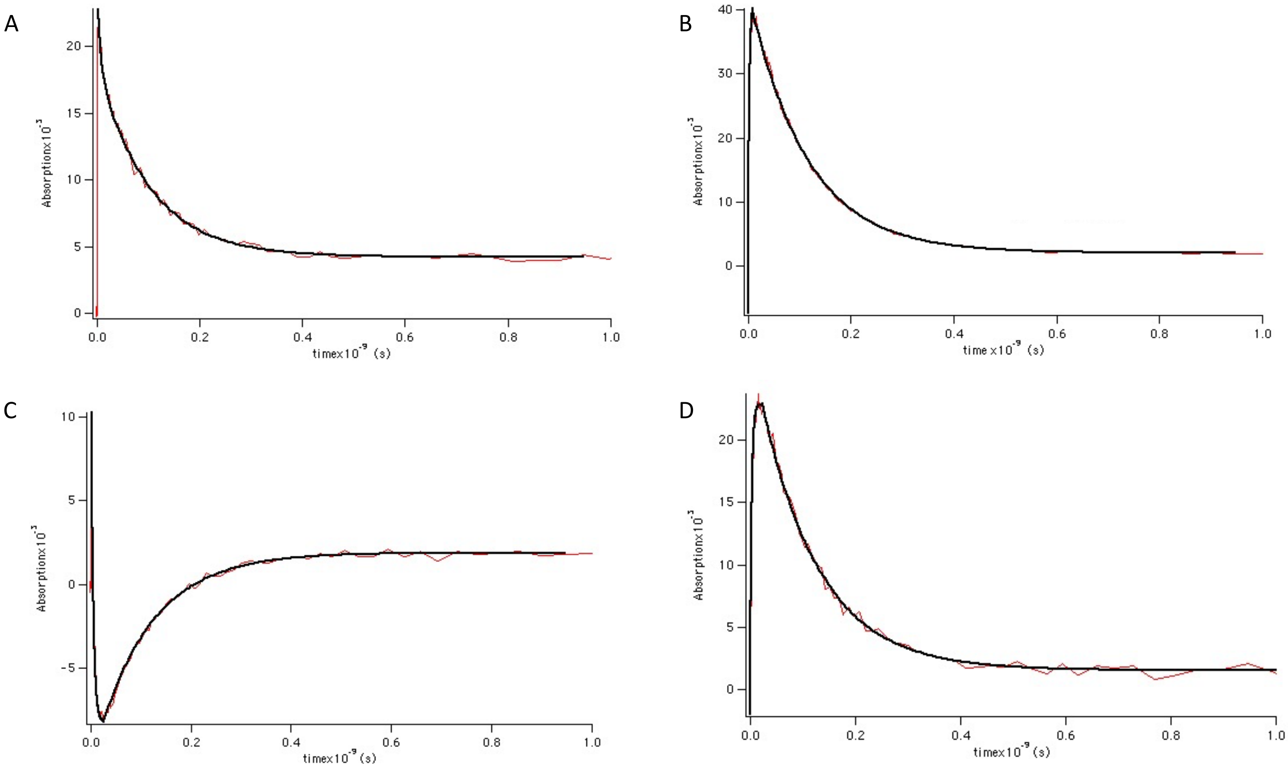

Figure S5. Transient kinetics of **1b** in acetonitrile probed at (**A**) 361 nm, (**B**) 482 nm, (**C**) 540 nm and (**D**) 617 nm.

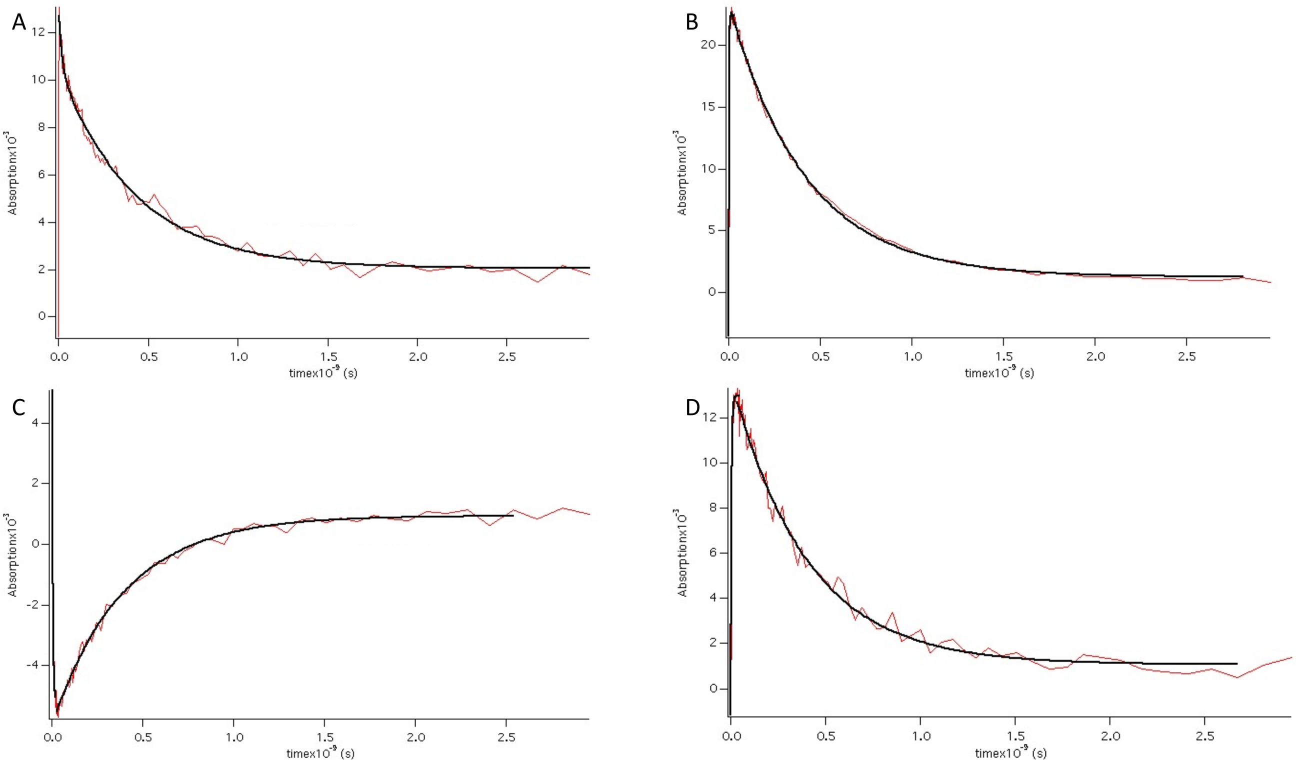

Figure S6. Transient kinetics of **1c** in acetonitrile probed at (**A**) 362 nm, (**B**) 481 nm, (**C**) 540 nm and (**D**) 616 nm.
